# *CYP* genetic variants and toxicity related to anti-tubercular agents: a systematic review and meta-analysis

**DOI:** 10.1186/s13643-018-0861-z

**Published:** 2018-11-20

**Authors:** Marty Richardson, Jamie Kirkham, Kerry Dwan, Derek J. Sloan, Geraint Davies, Andrea L. Jorgensen

**Affiliations:** 1 Department of Biostatistics, University of Liverpool, Liverpool, L69 3GB UK; 2 Cochrane Editorial Unit, London, SW1Y 4QX UK; 3 School of Medicine, University of St Andrews, St Andrews, KY16 9TF UK; 4 Department of Clinical Infection, Microbiology and Immunology, University of Liverpool, Liverpool, L69 3GB UK

**Keywords:** Tuberculosis, Pharmacogenetics, Toxicity, Meta-analysis

## Abstract

**Background:**

Treatment with anti-tuberculosis drugs may cause patients to experience serious adverse effects. Genetic factors, such as polymorphisms of *CYP* genes, may increase the likelihood of a patient experiencing such adverse drug reactions. In this systematic review and meta-analysis, we synthesised evidence for associations between *CYP* genetic variants and anti-tuberculosis drug-related toxicity outcomes.

**Methods:**

We searched MEDLINE, PubMed, EMBASE, BIOSIS and Web of Science to identify relevant studies. We performed meta-analyses to obtain an effect estimate for each genetic variant on each outcome, and stratified all analyses by country. We qualitatively assessed the methodological quality of the included studies.

**Results:**

We included data from 28 distinct cohorts of patients in the review. We identified many areas of concern with regard to the quality of included studies. Patients with homozygous mutant-type or heterozygous genotype at the *CYP2E1 Rsa*I polymorphism were significantly less likely to experience hepatotoxicity than patients with homozygous wild-type genotype (odds ratio [OR] = 0.75, 95% confidence interval [CI] 0.56–1.00; *p* = 0.047, *I*^2^ = 58.2%). No significant differences were observed for the *CYP2E1 Dra*I and *Pst*I polymorphisms. For the 96-bp deletion-insertion single-nucleotide polymorphism (SNP) of the *CYP2E1* gene, homozygous mutant-type significantly increased hepatotoxicity risk compared with homozygous wild-type (OR = 8.20, 95% CI 1.38–48.68, *I*^2^ = 0%); no significant difference was observed for heterozygous genotype compared with homozygous wild-type (OR = 0.77, 95% CI 0.19–3.21, *I*^2^ = 0%).

**Conclusions:**

Generally, we identified that coverage of the association between SNPs of *CYP* genes and anti-tuberculosis drug-related toxicity outcomes is incomplete. We observed significant associations between the *Rsa*I and 96-bp deletion-insertion SNPs of the *CYP2E1* gene and anti-tuberculosis drug-related hepatotoxicity. We were unable to comment on the impact of ethnicity on the investigated associations, as information on participants’ ethnicity was sparsely reported in the included studies.

**Systematic review registration:**

PROSPERO registration number: CRD42017068448.

**Electronic supplementary material:**

The online version of this article (10.1186/s13643-018-0861-z) contains supplementary material, which is available to authorized users.

## Background

Tuberculosis (TB) is an infectious disease caused by *Mycobacterium tuberculosis* bacteria and is the second most common cause of death from an infectious disease in adults (HIV/AIDS being the first) [1, 2]. For individuals with drug-susceptible TB, the World Health Organisation currently recommends combination treatment with four first-line drugs: isoniazid, rifampicin, ethambutol and pyrazinamide [1].

Treatment with anti-TB drugs may cause patients to experience serious adverse effects, such as anti-TB drug-induced hepatotoxicity (ATDH). Incidence rates of ATDH for patients treated with the standard combination treatment have been reported to vary from 2 to 28%, depending on the treatment regimen, patient characteristics (e.g. age, race and sex) and definition of ATDH [3]. ATDH may be fatal, with reported mortality rates of 6–12% if treatment is not stopped promptly [4]. ATDH and other anti-TB drug-related toxicity outcomes may also lead to poor patient adherence, which in turn may result in treatment failure, relapse and the emergence of drug resistance [3].

Proposed genetic risk factors for ATDH include polymorphisms of the Cytochrome P450 (*CYP*) genes. *CYP* genes encode the drug-metabolising CYP enzymes [5]. Therefore, *CYP* gene polymorphisms may affect CYP enzyme activity, altering the metabolic pathway of anti-TB drugs in the liver. Consequently, hepatic adverse reactions may occur. Toxic metabolites may also cause other adverse reactions, such as maculopapular eruption (MPE), although hepatotoxicity is the most widely studied outcome in pharmacogenetic studies of anti-TB drugs.

Isoniazid is the anti-TB drug for which mechanisms of the genetic contribution to ATDH have been most widely studied. In the liver, isoniazid is first metabolised into acetylisoniazid via *N*-acetyltransferase 2, followed by hydrolysis to acetylhydrazine [6]. Acetylhydrazine is proposed to be oxidised into hepatotoxic intermediates by CYP2E1 [7]. Therefore, variants of the *CYP2E1* gene may be associated with isoniazid-related hepatotoxicity, as CYP2E1 is one of the main enzymes involved in the metabolism of isoniazid [5].

Rifampicin and pyrazinamide have also been reported to cause hepatotoxicity [8], although the biological mechanisms for rifampicin- and pyrazinamide-induced hepatotoxicity remain unknown [9]. The OATP1B1*15 haplotype has been reported to be an important risk factor for rifampicin-induced liver injury [10]. No research into genetic risk factors for pyrazinamide-induced hepatotoxicity has been reported [11]. Ethambutol has not previously been reported to cause hepatotoxicity [8].

The aim of this systematic review and meta-analysis was to evaluate the current evidence for associations between *CYP* genetic variants and anti-TB drug-related toxicity. Meta-analyses investigating the association between *CYP2E1* genetic variants and hepatotoxicity have previously been published [12–16]. However, these meta-analyses have produced some conflicting results. For example, Wang et al. [16] identified that the *CYP2E1* c1/c1 genotype significantly increases the risk of ATDH (odds ratio [OR] = 1.32, 95% confidence interval [CI] 1.03–1.69, *I*^2^ = 55.9%%); however, Cai et al. [12] found no significant association for this same comparison (OR = 1.28, 95% CI 0.97–1.69, *I*^2^ = 34.2%). Three other published meta-analyses [13–15] identified a significant association between the *CYP2E1* c1/c1 genotype and increased risk of ATDH, although the pooled ORs reported by these meta-analyses ranged from 1.36 to 2.22. Furthermore, the previously conducted reviews have the following limitations:

Cai et al. [12], Deng et al. [13], Sheng et al. [14] and Wang et al. [16] all excluded studies if data required for meta-analysis were not included in the study report.

- Cai et al. [12] excluded three studies that were non-randomised controlled trials (RCTs), and Deng et al. [13], Sheng et al. [14] and Sun et al. [15] all included only case–control studies. Important evidence may have been omitted from these reviews, as pharmacogenetic data may be reported in RCTs, case–control studies or cohort studies.
- Cai et al. [12] did not assess the methodological quality of included studies. The other previously conducted meta-analyses used a checklist developed by Little et al. [17] to assess study quality.
- None of the previously conducted meta-analyses aimed to identify and synthesise data for *CYP* genetic variants other than *CYP2E1* genetic variants, or for outcomes other than hepatotoxicity; such exclusions may limit evidence-based recommendations.

We planned to overcome these limitations in our systematic review by: contacting study authors to obtain data required for meta-analysis when it was not included in the study report; including relevant studies regardless of their design; and performing a rigorous quality assessment of included studies. In addition, we did not exclude studies that did not report hepatotoxicity, and we aimed to identify and synthesise data for all *CYP* genetic variants. Therefore, the scope of our review is wider than the previously conducted meta-analyses.

## Methods

The current study forms part of a series of systematic reviews and meta-analyses evaluating the influence of different genetic variants on toxicity to anti-TB agents, the protocol for which has been published (PROSPERO registration number: CRD42017068448) [18]. This review has been conducted in accordance with the PRISMA statement [19]; a completed copy of the PRISMA checklist is provided in Additional file 1.

The described search strategy and study selection methods were used to identify studies that investigated the effect of any genetic variant (rather than specifically *CYP* genetic variants) on anti-TB drug-related toxicity. However, in this article, we focus only on studies that reported data for the association between *CYP* variants and anti-TB drug-related toxicity outcomes. Studies investigating associations between other genetic variants and anti-TB drug-related toxicity will be reported separately.

### Selection criteria

#### Types of studies

Eligible study designs were cohort studies, case–control studies and RCTs.

#### Types of participants

We included studies that recruited TB patients who were either already established on anti-TB treatment or were commencing treatment (at least one of isoniazid, rifampicin, pyrazinamide or ethambutol), and who had been genotyped, in order to investigate the association between genetic variant(s) and anti-TB drug-related toxicity outcomes. Specifically, we only included studies where over 50% of included patients were TB patients receiving anti-TB treatment, as we would then contact study authors to obtain data for the subgroup of TB patients, as suggested in the Cochrane Handbook [20] for studies where only a subset of the population is eligible.

#### Types of outcomes

Studies that measured any anti-TB drug-related toxicity outcomes were eligible for inclusion.

### Search strategy

An information specialist (Eleanor Kotas) designed the search strategy (provided in Additional file 2). MEDLINE, PubMed, EMBASE, BIOSIS and Web of Science were searched for relevant studies. We hand searched the reference lists of relevant studies, and contacted experts in the clinical area to identify further eligible studies. Only studies published in English were included, but we did not restrict by year of publication or by publication status.

### Study selection

We imported the results of the search into Covidence [21]. One author (MR) removed duplicates and scanned the study abstracts to remove obviously irrelevant studies. A second author (ALJ, JK or KD) independently screened a sample of 10% of studies.

One reviewer (MR) obtained the full text for each potentially relevant study and assessed eligibility based on the eligibility criteria. A second author (ALJ, JK or KD) independently screened a sample of 10% of studies for inclusion. Any disagreements between the two reviewers at both the abstract and full-text screening stages were resolved through discussion, or by consulting a third author if necessary.

### Outcomes

The primary outcome of our review was hepatotoxicity by any definition used by the original investigators. The secondary outcomes were all other toxicity outcomes reported in the included studies.

### Data extraction

We pre-piloted a data extraction form, which was designed to enable collection of data on study design, participant characteristics, treatment regimen, genotype groups and outcomes. One author (MR) extracted data, following methods described in the Cochrane Handbook [20] and The HuGENet HuGE Review Handbook [22]. A second author (ALJ, JK or KD) independently extracted all outcome data. Any disagreements between the two reviewers were resolved through discussion, or by consulting a third author if necessary. We contacted study authors if outcome data required for meta-analysis were not included in the study report.

We examined author lists, locations, dates of recruitment and other study characteristics to identify cases of multiple articles reporting data for overlapping or identical patient cohorts. If we suspected that this may be the case, we contacted authors to clarify whether patient cohorts were distinct. If an author clarified that multiple articles reported outcomes for the same patient cohort, or overlapping cohorts, or if we suspected this based on reported study characteristics, we assigned a group identifier (GI) to these articles. Assigning this GI ensured that data for each patient cohort were only included once in any meta-analysis.

### Quality assessment

One author (MR) used the criteria developed by Jorgensen and Williamson [23] specifically for pharmacogenetic studies, to assess the methodological quality of each included study. A second author (ALJ) independently assessed the quality of a sample of 10% of studies. Any disagreements between the two reviewers were resolved through discussion. We summarised the number of studies meeting each criterion in the text.

### Data synthesis

#### Primary analyses

The primary analyses assessed the risk of hepatotoxicity in individuals with homozygous mutant genotype or heterozygous genotype and compared it with the risk in those with homozygous wild-type genotype for three key single-nucleotide polymorphisms (SNPs) of the *CYP2E1* gene, commonly known as the *Rsa*I, *Pst*I and *Dra*I polymorphisms; these SNPs are the most widely studied polymorphisms of *CYP* genetic variants in the context of ATDH. Data were pooled from studies that reported data for each genotype group separately with data from studies that combined homozygous mutant-type and heterozygous genotype groups.

For each SNP, sensitivity analyses were conducted to investigate the robustness of the primary analysis by performing pairwise comparisons of heterozygous versus homozygous wild-type genotype, and homozygous mutant versus homozygous wild-type genotype. For these analyses, it was only possible to include data from studies that reported on each genotype group separately.

We produced funnel plots for each of the primary analyses (where at least ten studies were included) to investigate the possibility of publication bias.

#### Secondary analyses

The secondary analyses investigated all other associations between *CYP* genetic variants and anti-TB drug-related toxicity outcomes. We performed meta-analyses for all associations that were investigated by at least two studies:

- For SNPs where all studies presented data for each genotype group separately, we performed two pairwise comparisons; heterozygous genotype versus homozygous wild-type and homozygous mutant-type versus homozygous wild-type.
- For SNPs where all studies presented data for combined genotype groups, we performed one comparison of the combined genotype groups.
- For SNPs where the approach varied between studies, we pooled data for studies that reported data for each genotype group separately with data from studies that reported data for combined genotype groups. We also performed pairwise comparisons including data from studies that reported on each genotype group separately.

For SNPs investigated by one study only, ORs comparing genotype groups were calculated and summarised with their 95% CIs in a table, together with the pooled estimates from all meta-analyses.

All meta-analyses were performed using the metan package in Stata 14 [24]. A random-effects model was employed because we anticipated heterogeneity between studies due to differences in study design, quality of methods, ethnic background of participants and outcome definitions. We assessed statistical heterogeneity by visually examining the forest plots, and by referring to the *I*^2^ statistic. The random-effects model used the method of DerSimonian and Laird [25], with the estimate of heterogeneity being taken from the Mantel–Haenszel model [26]. If no events occurred in one of the genotype groups, a continuity correction of 0.5 was applied [20]. If there were no patients in one of the genotype groups in a comparison for a particular study, data from this study were excluded from the meta-analysis.

According to the HuGENet HuGE Review Handbook, meta-analyses of genetic association studies should be stratified by ethnicity; results across different ethnic groups should only be pooled if effect estimates across these groups appear sufficiently similar [22]. Information on participants’ ethnicity was sparsely reported; however, in an attempt to adhere with this recommendation, we performed analyses stratified by the countries in which studies were conducted.

## Results

### Included and excluded studies

**Fig. 1:**
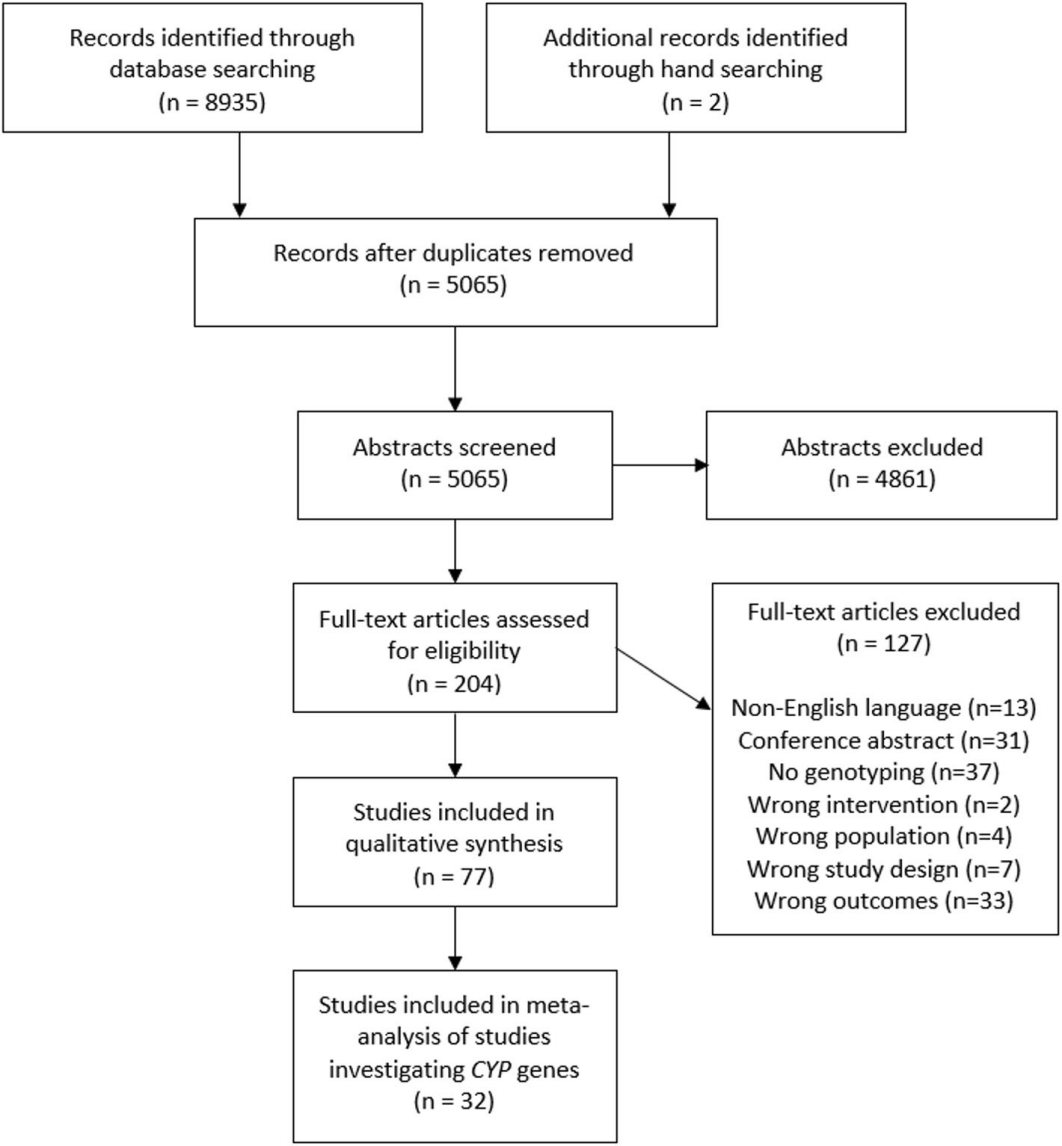
PRISMA flow diagram

A PRISMA flowchart, showing selection and elimination of studies during the literature search, is provided in Fig. 1. We included 77 articles, and identified 53 distinct cohorts of patients.

Thirty-three articles reported data for the association between *CYP* genetic variants and anti-TB drug-related toxicity; we identified 28 distinct cohorts of patients from these articles. We did not include data from one article [27], as we suspected that this article reported data for the same group of patients included in another paper [28]; furthermore, the data presented were unclear and we were unable to clarify the data with the authors. It was also excluded from our quality assessment. Therefore, in this review, we include data from 32 articles [28–59] (28 distinct patient cohorts). The characteristics of studies included in this review are provided in Additional file 3: Table S1.

### Quality assessment

#### Choosing which genes and SNPs to genotype

Twenty articles provided justification for the choice of gene and SNP to be investigated. For the 12 articles [31, 33, 36, 44, 46, 48, 52, 54–56, 58, 59] that did not provide justification for each investigated gene and SNP, no articles limited their reporting to only statistically significant associations. Therefore, selective reporting of genes and SNPs does not appear to be an issue of concern.

#### Sample size

The median sample size of included studies was 220.5 (interquartile range 155.5–332). Typically, much larger sample sizes are required to detect genetic effects [23]. Only one article [59] reported the a priori power to detect pre-specified effect sizes. Therefore, most studies are likely to be at risk of being underpowered [23].

#### Study design

Twelve articles used a case–control design [29, 35, 38, 39, 41, 42, 47, 50–53, 55]; however, only 1 article [39] reported that the 2 groups were genotyped in mixed batches. Separate genotyping in case–control studies could potentially bias the results of a study [23]. A total of 19 articles reported prospective cohort studies [28, 30–34, 36, 37, 40, 43–46, 48, 49, 54, 56–58], and 1 article reported a retrospective cohort study [59].

#### Reliability of genotypes

Only 5 articles [34, 39, 50–52] mentioned genotype quality control procedures, and therefore 27 articles may be at risk of incorrect genotype allocation [23]. Only five articles [37, 38, 44, 46, 49] compared genotype frequencies of all investigated SNPs to previously published genotype frequencies for the same population. Such a simple check can be an effective method of identifying problems with genotyping. For case–control and retrospective cohort studies, genotyping personnel should be blinded to outcome status in order to minimise the risk of bias during the genotyping procedure [23]. However, only 4 articles [39, 50–52] of 13 case–control/retrospective cohort studies mentioned that genotyping personnel were blinded to outcome status.

#### Missing genotype data

For most articles (19/32, 59.4%), the number of participants included in the analyses was the same as the study sample size, so it was clear that there were no missing genotype data. For the remaining 13 articles [34, 38, 41–43, 46, 47, 50, 51, 53, 58, 59], only 6 articles [34, 38, 47, 53, 58, 59] summarised the extent of missing data for all genes and SNPs analysed. No articles described checking whether missing data were missing at random; therefore, 13 articles are at risk of bias from non-random missing data [23].

#### Population stratification

Two articles [36, 46] conducted tests to detect population stratification, but did not identify any population stratification. One article applied a strict exclusion criterion, which ensured that included patients were from a non-diverse ethnic group [48]. All other studies are at risk of confounding due to population stratification.

#### Hardy–Weinberg equilibrium

Testing for deviation from the Hardy–Weinberg equilibrium (HWE) can highlight genotyping errors, population stratification and other problems [23]. Sixteen articles [28, 34–37, 40–42, 46, 50–52, 56–59] tested for HWE for all SNPs investigated, and a further 3 [29, 44, 53] tested for HWE for a subset of SNPs. The remaining 13 articles did not report on HWE testing.

#### Mode of inheritance

Nineteen articles assumed a specific underlying mode of inheritance [29, 31, 32, 35, 37, 39, 42, 43, 45–47, 49, 53–59]. Three of these 19 articles detailed their reasoning behind this assumption [32, 47, 56]; for the remaining 16 articles, several analyses assuming different modes of inheritance may have been performed, with only the most statistically significant being reported [23]. There is therefore a risk of selective reporting within these 16 articles. Three articles [41, 50, 51] conducted analyses assuming different modes of inheritance, but only one of these articles [41] adjusted their analyses for multiple testing; therefore, there is a risk of an inflated type I error rate in the other two articles.

#### Choice and definition of outcomes

Definitions of hepatotoxicity (Additional file 4: Table S2) varied considerably between the included studies. Of the 30 articles reporting data for this outcome, 1 article did not define hepatotoxicity [45], 1 provided a vague definition [39] and the remaining 28 articles provided 22 different definitions.

Definitions of other toxicity outcomes reported are provided in Additional file 5: Table S3. These definitions were generally not sufficiently detailed to assess how similar they were to each other.

Twenty-eight articles all provided justification for the choice of outcomes. Four articles [30, 34, 53, 59] did not provide justification for the choice of outcomes, but the choice of outcomes was appropriate to address the main study aim as described in the article introduction. Therefore, there is no evidence to suggest that selective reporting of outcomes is an issue of concern for the included articles.

#### Treatment adherence

Only three articles [33, 34, 42] reported that treatment adherence was assessed. For two of these articles, it was not necessary to adjust for adherence in the analyses, as patients were reported to have good treatment adherence [33, 34]. The third article that assessed treatment adherence excluded patients who did not adhere to treatment [42]. One article [48] reported that anti-TB drugs were administered by directly observed therapy, short-course, so it was unnecessary to assess adherence.

### Association between *CYP* genetic variants and anti-TB drug-related toxicity

Data from 28 distinct cohorts across the 32 included papers were considered for the analyses of association reported below.

#### Primary analyses: key *CYP2E1* SNPs and hepatotoxicity

**Fig. 2:**
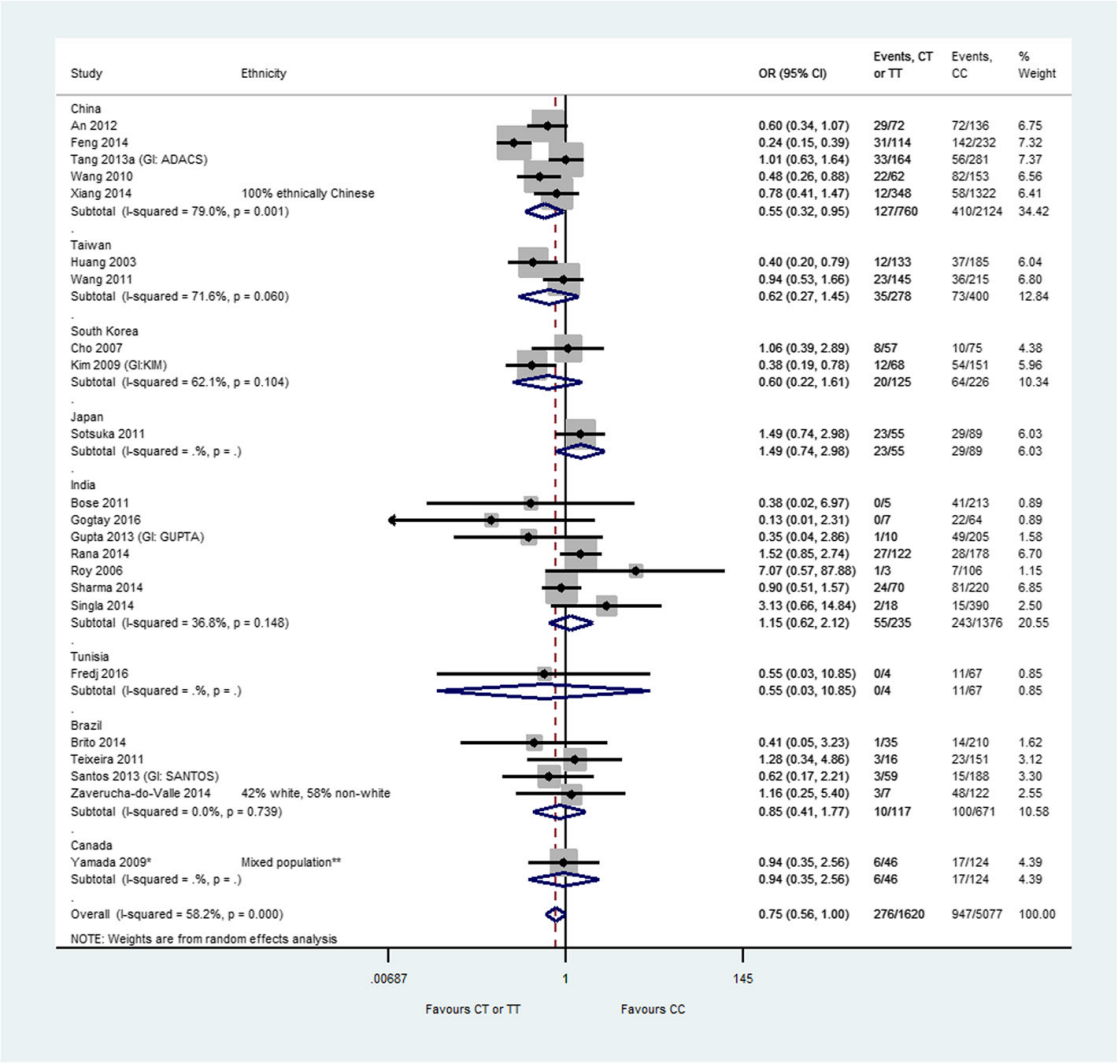
*CYP2E1 Rsa*I polymorphism and anti-tuberculosis drug-induced hepatotoxicity: homozygous mutant-type (TT) or heterozygous genotype (CT) versus homozygous wild-type (CC). The labels on this graph indicate which genotype group is favoured, i.e. the likelihood of hepatotoxicity is reduced in the favoured genotype group. * Yamada 2009 was conducted in the latent TB population. **Asian: 72 (42%), Caucasian: 49 (29%), South Asian: 22 (13%), Hispanic: 7 (4%), Middle Eastern: 8 (5%), First Nations: 5 (3%), Other/mixed/unknown: 7 (4%). *CI* confidence interval, *GI* group identifier, *OR* odds ratio

**Fig. 3:**
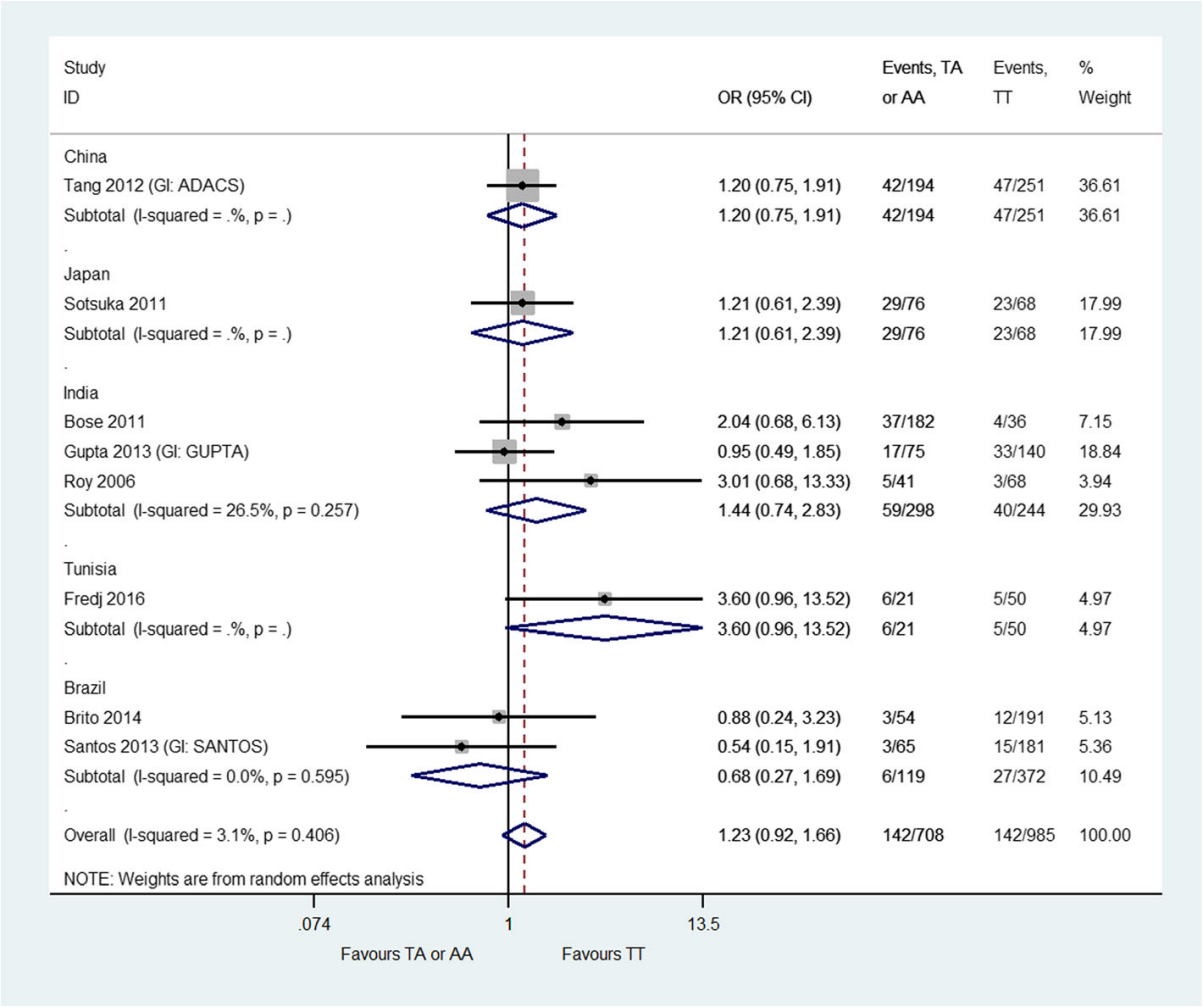
*CYP2E1 Dra*I polymorphism and anti-tuberculosis drug-induced hepatotoxicity: homozygous mutant-type (AA) or heterozygous genotype (AT) versus homozygous wild-type (TT). None of the included studies reported ethnicity so this information is not provided on the forest plot. The labels on this graph indicate which genotype group is favoured i.e. the likelihood of hepatotoxicity is reduced in the favoured genotype group. *CI* confidence interval, *GI* group identifier, *OR* odds ratio

**Fig. 4:**
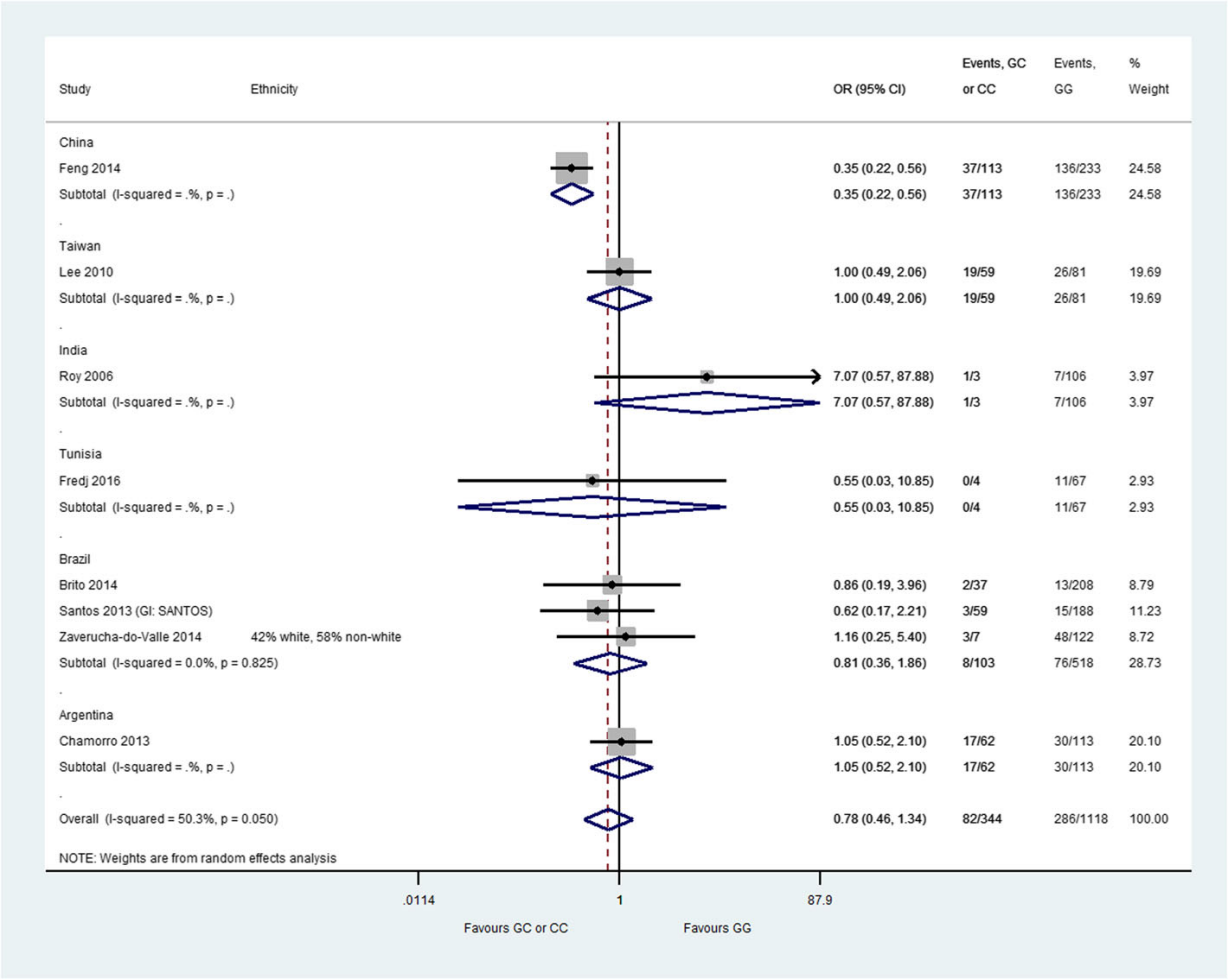
CYP2E1 *Pst*I polymorphism and anti-TB drug-induced hepatotoxicity: homozygous mutant-type (CC) or heterozygous genotype (GC) versus homozygous wild-type (GG). The labels on this graph indicate which genotype group is favoured i.e. the likelihood of hepatotoxicity is reduced in the favoured genotype group. *CI* confidence interval, *GI* group identifier, *OR* odds ratio

Forest plots displaying the results of the primary analyses are provided in Figs. 2, 3 and 4.

Patients with TT or CT genotype at the *CYP2E1 Rsa*I polymorphism were significantly less likely (*p* = 0.047) to experience hepatotoxicity than patients with CC genotype (OR = 0.75, 95% CI 0.56–1.00) (Fig. 2). Moderate heterogeneity was observed in this analysis *(I*^2^ = 58.2%). The results of the sensitivity analyses are provided in Additional file 6. For the *CYP2E1 Rsa*I polymorphism, no significant differences were observed for either pairwise comparison (heterozygous genotype versus homozygous wild-type: OR = 0.80, 95% CI 0.58–1.10, *I*^2^ = 48.4%; homozygous mutant-type versus homozygous wild-type: OR = 1.03, 95% CI 0.68–1.55, *I*^2^ = 2.7%).

There was no significant difference in the risk of hepatotoxicity between patients with AA or TA genotype at the *CYP2E1 Dra*I polymorphism and patients with TT genotype (OR = 1.23, 95% CI 0.92–1.66) (Fig. 3). Minimal heterogeneity was observed in this analysis (*I*^2^ = 3.1%). The sensitivity analyses (two pairwise comparisons) also showed no significant differences between genotype groups (heterozygous genotype versus homozygous wild-type: OR = 1.28, 95% CI 0.93–1.77, *I*^2^ = 6.4%; homozygous mutant-type versus homozygous wild-type: OR = 1.34, 95% CI 0.57–3.16, *I*^2^ = 29.4%) (Additional file 6).

There was no significant difference in the risk of hepatotoxicity between patients with CC or GC genotype at the *CYP2E1 Pst*I polymorphism and patients with GG genotype (OR = 0.78, 95% CI 0.46–1.34) (Fig. 4). Moderate heterogeneity was observed in this analysis (*I*^2^ = 50.3%). The sensitivity analyses (two pairwise comparisons) also showed no significant differences between genotype groups (heterozygous genotype versus homozygous wild-type: OR = 1.05, 95% CI 0.66–1.65, *I*^2^ = 0%; homozygous mutant-type versus homozygous wild-type: OR = 1.04, 95% CI 0.36–2.99, *I*^2^ = 0%) (Additional file 6).

The heterogeneity observed in the analyses for the *Rsa*I and *Pst*I polymorphisms may be due to the variable distribution of genotypes in different geographic areas, which we assumed to be a proxy for ethnic group. Owing to the small numbers of studies conducted in each country, it is difficult to draw firm conclusions from the stratified analyses about the effect of country on the investigated genetic associations.

We produced a funnel plot for each of the primary analyses (Additional file 7). There was no evidence to suggest that publication bias was an issue of concern.

#### Secondary analyses: *CYP* genetic variants and hepatotoxicity

The included studies reported data for 8 *CYP* genes and 24 SNPs (in addition to the *CYP2E1* SNPs reported in the primary analyses). A summary of all data for the association between *CYP* genetic variants and hepatotoxicity is provided in Table 1. There were sufficient data to perform meta-analyses for three SNPs, and forest plots showing the results of these meta-analyses are provided in Additional file 8. The findings from these meta-analyses are:

- For the 96-bp deletion-insertion SNP of the *CYP2E1* gene, homozygous mutant-type significantly increases hepatotoxicity risk compared with homozygous wild-type (OR = 8.20, 95% CI 1.38–48.68, *I*^2^ = 0%), but no significant difference was observed for heterozygous genotype compared with homozygous wild-type (OR = 0.77, 95% CI 0.19–3.21, *I*^2^ = 0%).
- For the rs4918758 SNP of the *CYP2C9* gene, no significant differences were observed for either pairwise comparison (heterozygous genotype versus homozygous wild-type: OR = 1.11; 95% CI 0.53–2.31, *I*^2^ = 66.7%; homozygous mutant-type versus homozygous wild-type: OR = 0.87, 95% CI 0.51–1.50, *I*^2^ = 0%). The heterogeneity observed in the heterozygous genotype versus homozygous wild-type comparison may be due to the variable distribution of genotypes in different geographic areas.
- For the rs3745274 SNP of the *CYP2B6* gene, no significant differences were observed for either pairwise comparison (heterozygous genotype versus homozygous wild-type: OR = 1.49, 95% CI 0.87–2.55, *I*^2^ = 0%; homozygous mutant-type versus homozygous wild-type: OR = 1.51, 95% CI 0.55–4.13, *I*^2^ = 4.2%).
- Due to the small numbers of studies conducted in each country, it is difficult to draw firm conclusions from the stratified analyses about the effect of country on the investigated genetic associations Additional files 3, 4 and 5.

**Table 1:** Results of the secondary analyses: association between CYP genetic variants and hepatotoxicity

Gene	Variant	Comparison	Country (no. of studies)	Ethnicity	OR (95% CI)	# cases	# controls	*I*^2^ value
CYP2E1	Rs2080672	Het (AG) vs Hom WT (AA)	China (1 study)	NR	1.16 (0.72, 1.89)	86	334	N/A
		Hom MT (GG) vs Hom WT (AA)	China (1 study)	NR	0.69 (0.19, 2.42)	54	228	N/A
	Rs915908	Het (GA) vs Hom WT (GG)	China (1 study)	NR	0.89 (0.47, 1.69)	79	318	N/A
		Hom MT (AA) vs Hom WT (GG)	China (1 study)	NR	1.09 (0.52, 2.32)	75	292	N/A
	Rs8192775	Het (GA) vs Hom WT (GG)	China (1 study)	NR	1.17 (0.72, 1.90)	85	333	N/A
		Hom MT (AA) vs Hom WT (GG)	China (1 study)	NR	0.76 (0.25, 2.29)	55	234	N/A
	Rs2515641	Het (CT) vs Hom WT (CC)	China (1 study)	NR	1.20 (0.73, 1.99)	85	342	N/A
		Hom MT (TT) vs Hom WT (CC)	China (1 study)	NR	1.31 (0.41, 4.18)	60	252	N/A
	Rs2515644	Het (CA) vs Hom WT (CC)	China (1 study)	NR	1.26 (0.74, 2.15)	73	285	N/A
		Hom MT (AA) vs Hom WT (CC)	China (1 study)	NR	1.04 (0.52, 2.08)	42	186	N/A
	Rs2070672	Het (AG) vs Hom WT (AA)	South Korea (1 study)	NR	1.74 (0.93, 3.25)	63	149	N/A
		Hom MT (GG) vs Hom WT (AA)	South Korea (1 study)	NR	0.94 (0.18, 4.85)	41	116	N/A
	Rs2070673^a^	Het (TA) vs Hom WT (TT)	South Korea (1 study)	NR	0.88 (0.48, 1.63)	59	134	N/A
		Hom MT (AA) vs Hom WT (TT)	South Korea (1 study)	NR	0.75 (0.28, 1.96)	37	84	N/A
	96-bp (deletion-insertion SNP)	Het (DI) vs Hom WT (DD)	India (1 study)	NR	1.13 (0.22, 5.88)	6	98	N/A
			Brazil (1 study)	NR	0.25 (0.01, 4.26)	18	228	N/A
			All (2 studies)		*0*.*77* (*0*.*19*, *3*.*21*)	*24*	*326*	*0*.*0*%
		Hom MT (II) vs Hom WT (DD)	India (1 study)	NR	11.56 (1.37, 97.67)	5	55	N/A
			Brazil (1 study)	NR	3.72 (0.15, 94.60)	18	207	N/A
			All (2 studies)		*8*.*20* (*1*.*38*, *48*.*68*)	*23*	*262*	*0*.*0*%
CYP2C9	Rs4918758^b^	Het (TC) vs Hom WT (TT)	China (1 study)	NR	0.78 (0.46, 1.33)	69	285	N/A
			South Korea (1 study)	NR	1.66 (0.85, 3.23)	59	127	N/A
			All (2 studies)		*1*.*11* (*0*.*53*, *2*.*31*)	*128*	*412*	*66*.*7*%
		Hom MT (CC) vs Hom WT (TT)	China (1 study)	NR	0.94 (0.49, 1.80)	51	188	N/A
			South Korea (1 study)	NR	0.72 (0.27, 1.95)	24	80	N/A
			All (2 studies)		*0*.*87* (*0*.*51*, *1*.*50*)	*75*	*268*	*0*.*0*%
	Rs9332098	Het (GA) vs Hom WT (GG)	China (1 study)	NR	0.32 (0.07, 1.38)	88	354	N/A
		Hom MT (AA) vs Hom WT (GG)	China (1 study)	NR	Data excluded^c^			
	Rs9332096	Het (CT) vs Hom WT (CC)	South Korea (1 study)	NR	0.63 (0.27, 1.47)	66	156	N/A
		Hom MT (TT) vs Hom WT (CC)	South Korea (1 study)	NR	0.73 (0.03, 18.24)	58	129	N/A
	Rs1057910	Het (AC) vs Hom WT (AA)	South Korea (1 study)	NR	1.00 (0.34, 2.97)	64	154	N/A
		Hom MT (CC) vs Hom WT (AA)	South Korea (1 study)	NR	Data excluded^c^			
CYP2B6	rs3745274	Het (GT) vs Hom WT (GG)	Brazil (1 study)	NR	1.57 (0.71, 3.45)	30	176	N/A
			Ethiopia (1 study)	NR	1.42 (0.68, 2.98)	35	145	N/A
			All (2 studies)		*1*.*49* (*0*.*87*, *2*.*55*)	*65*	*321*	*0*.*0*%
		Hom MT (TT) vs Hom WT (GG)	Brazil (1 study)	NR	0.58 (0.07, 4.81)	13	103	N/A
			Ethiopia (1 study)	NR	1.98 (0.66, 5.87)	22	94	N/A
			All (2 studies)		*1*.*51* (*0*.*55*, *4*.*13*)	*35*	*197*	*4*.*2*%
CYP3A4	rs12333983	Het (TA) vs Hom WT (TT)	China (1 study)	NR	1.33 (0.81, 2.18)	78	312	N/A
		Hom MT (AA) vs Hom WT (TT)	China (1 study)	NR	1.33 (0.62, 2.86)	47	204	N/A
	-392 A-G	Het (GA) vs Hom WT (AA)	Brazil (1 study)	42% white, 58% non-white	0.69 (0.32, 1.47)	45	69	N/A
		Hom MT (GG) vs Hom WT (AA)	Brazil (1 study)	42% white, 58% non-white	0.91 (0.31, 2.70)	34	45	N/A
CYP2C19	rs11568732	Het (TG) vs Hom WT (TT)	China (1 study)	NR	0.54 (0.25, 1.19)	87	350	N/A
		Hom MT (GG) vs Hom WT (TT)	China (1 study)	NR	0.93 (0.10, 8.47)	80	229	N/A
	rs4986894	Het (TC) vs Hom WT (TT)	China (1 study)	NR	0.95 (0.57, 1.59)	72	302	N/A
		Hom MT (CC) vs Hom WT (TT)	China (1 study)	NR	1.11 (0.53, 2.32)	48	191	N/A
	rs17878465	Het (CT) vs Hom WT (CC)	South Korea (1 study)	NR	0.99 (0.50, 1.94)	65	153	N/A
		Hom MT (TT) vs Hom WT (CC)	South Korea (1 study)	NR	0.33 (0.02, 6.58)	49	118	N/A
	rs4986893	Het (GA) vs Hom WT (GG)	South Korea (1 study)	NR	0.69 (0.31, 1.56)	66	156	N/A
		Hom MT (AA) vs Hom WT (GG)	South Korea (1 study)	NR	0.74 (0.03, 18.42)	57	128	N/A
CYP3A5	rs776746	Het (AG) vs Hom WT (AA)	Brazil (1 study)	NR	1.84 (0.83, 4.05)	31	189	N/A
		Hom MT (GG) vs Hom WT (AA)	Brazil (1 study)	NR	Data excluded^c^			
	Number of CYP3A5*1	One copy vs zero copies	Ethiopia (1 study)	NR	1.56 (0.76, 3.20)	39	151	N/A
		Two copies vs zero copies	Ethiopia (1 study)	NR	1.02 (0.21, 5.05)	24	110	N/A
CYP1A1	MspI	Hom MT or Het vs Hom WT	China (1 study)	NR	1.33 (0.81, 2.19)	127	127	N/A
CYP2D6	rs1080983	Het (GA) vs Hom WT (AA)	South Korea (1 study)	NR	0.83 (0.43, 1.61)	65	152	N/A
		Hom MT (GG) vs Hom WT (AA)	South Korea (1 study)	NR	0.56 (0.06, 5.11)	50	113	N/A
	rs1080989	Het (GA) vs Hom WT (AA)	South Korea (1 study)	NR	0.89 (0.45, 1.74)	50	121	N/A
		Hom MT (GG) vs Hom WT (AA)	South Korea (1 study)	NR	1.03 (0.47, 2.27)	36	80	N/A

*CI* confidence interval, *Het* heterozygous genotype, *Hom MT* homozygous mutant-type, *Hom WT* homozygous wild-type, *N/A* not applicable, *NR* not reported, *OR* odds ratio

^a^The paper (Kim 2009 [GI: KIM]) reports WT to be A and MT to be T, but data suggest that WT is T and MT is A

^b^One of the studies (Kim 2009 [GI: KIM]) reports WT to be C and MT to be T, but the other study (Tang 2013b [GI: ADACS]), and the data, suggest that WT is T and MT is C

^c^Data excluded due to zero counts in one of the genotype groups

The italicised values are pooled results from more than one study, i.e. the results of meta-analyses

### Secondary analyses: *CYP* genetic variants and other toxicity outcomes

A summary of all data for the association between *CYP* genetic variants and toxicity outcomes (other than hepatotoxicity) is provided in Additional file 9: Table S4. It was not possible to perform meta-analyses for any toxicity outcomes other than hepatotoxicity as there were no comparisons for which more than one study provided data, so each reported result is based on data from a single study.

Considering the impact of the *CYP2E1 Dra*I polymorphism on the outcome of “adverse drug-induced hepatotoxicity outcome” (definition unclear, this was reported as a separate outcome to ATDH), no significant association was reported for homozygous mutant-type or heterozygous genotype versus homozygous wild-type. For the outcome of anti-TB drug (ATD)-induced MPE, no significant associations were observed for any of the three investigated SNPs of the *CYP2E1* gene (*Rsa*I, rs2070672, rs2070673), for two SNPs of the *CYP2C9* gene (rs4918758, rs1057910) and for one SNP of the *CYP2C19* gene (-1418 C-T). For the rs9332096 SNP of the *CYP2C9* gene and the rs4986893 SNP of the *CYP2C19* gene, homozygous mutant-type or heterozygous genotype was found to significantly decrease the likelihood of ATD-induced MPE compared with homozygous wild-type (rs9332096: OR = 0.23, 95% CI 0.07–0.78; rs4986893: OR = 0.30, 95% CI 0.10–0.88).

## Discussion

### Meta-analyses

Where possible, we synthesised the results of the included studies in meta-analyses. Three [13, 14, 16] of the five previously mentioned meta-analyses performed analyses for the *Rsa*I and *Pst*I polymorphisms combined, presumably because these polymorphisms have been reported to be in linkage disequilibrium [60]. The approach taken for the analysis of *CYP2E1* polymorphisms in the other two meta-analyses was unclear. However, we identified studies reporting data for these two polymorphisms separately [31, 35], so we performed separate meta-analyses for each polymorphism.

We found that patients with homozygous wild-type (TT) or heterozygous (CT) genotype at the *CYP2E1 Rsa*I polymorphism were significantly less likely to experience hepatotoxicity than patients with CC genotype (OR = 0.75, 95% CI 0.56–1.00; *p* = 0.047). This result is consistent with the findings of four previously conducted meta-analyses [13, 14, 16]. In general, the plausibility of the findings for a significant association between the *CYP2E1 Rsa*I polymorphism and ATDH is well supported by the theory that CYP2E1 plays a role in the pathway of the metabolism of isoniazid in the liver [5], forming hepatotoxic intermediates [7].

We observed no significant association for the *CYP2E1 Dra*I polymorphism and ATDH, a result which is consistent with previous meta-analyses [14, 16]. We also observed no significant association for the *CYP2E1 Pst*I polymorphism and ATDH; this result is not consistent with the findings of previously conducted meta-analyses [13, 14, 16]. This may be because we only included studies that explicitly stated that results were for the *Pst*I polymorphism or the *Rsa*I/*Pst*I polymorphisms combined (if these alleles were in complete linkage disequilibrium), whereas the previously conducted meta-analyses do not mention using such an approach. The number of studies contributing data to the analysis of the *CYP2E1 Pst*I polymorphism was relatively small (*n* = 8) compared with the number of studies contributing data to the analysis of the *CYP2E1 Rsa*I polymorphism (*n* = 23).

We identified that for the 96-bp deletion-insertion SNP of the *CYP2E1* gene, homozygous mutant-type significantly increases hepatotoxicity risk compared with homozygous wild-type (OR = 8.20, 95% CI 1.38–48.68, *I*^2^ = 0%). To the best of our knowledge, no meta-analyses have been previously conducted for this variant. Furthermore, we are unaware of the publication of any other meta-analyses for SNPs of *CYP* genetic variants other than the *Rsa*I, *Dra*I and *Pst*I polymorphisms, so our results add to the existing understanding of the association between *CYP* genetic variants and hepatotoxicity.

### Quality assessment

We identified many areas of concern with regard to the quality of included studies. Most studies had considerably smaller sample sizes than would typically be required to provide power to detect a genetic association [23]. Furthermore, readers of almost all of the included studies would not be aware of the possibility of false-negative findings, due to the fact that only one study reported an a priori power calculation. We also had concerns about the possibility of incorrect genotype allocation in the included studies, as 84% of studies did not describe any genotyping quality control procedures. No studies described checking that missing data were randomly distributed. Any deviation from random missingness is a potential source of bias [23].

Most (91%) studies were at risk of potential bias due to population stratification. Furthermore, 41% of the studies did not report on testing of HWE, which can be useful for identifying genotyping errors, population stratification and other problems [23]. We noted that 50% of included studies may be at risk of selective reporting of analyses assuming different modes of inheritance, as these studies did not provide rationale for their selected mode of inheritance. Most of the included studies (88%) were at risk of bias from not adjusting for treatment adherence; the proportion of variability explained by genetic factors in these studies may be underestimated [23].

Although we identified methodological limitations of the included studies relating to some of the quality criteria, we did not identify any studies that were thought to be of particularly poor quality overall, so we did not consider it necessary to exclude any particular study in sensitivity analyses.

### Limitations

While conducting this systematic review and meta-analysis, we found that conducting robust synthesis of the existing evidence base is challenging, owing to variability between studies in terms of the genetic variants investigated, how participants are classified according to genotype, choice and definition of outcomes, ethnicity of participants and methodological quality. In order to address this variability, we performed meta-analyses stratified by genetic variants, genotype contrasts and outcomes. We also stratified further by the country in which the study was undertaken as a proxy variable for ethnicity, which was not widely reported.

Clearly, our approach of stratifying by country instead of by ethnicity is not ideal, as the population of any country is often ethnically diverse. However, multiple studies from a single country are likely to be relatively similar with regard to the ethnicity of participants, so stratifying by country was considered to be the most suitable alternative to stratifying by ethnicity. Consequently, we were unable to comment on the impact of ethnicity on the investigated associations; this is an important limitation of the review as the distribution of *CYP* alleles differs considerably between different ethnic populations [61].

Due to the number of references identified by the search strategy and the number of studies included in this review, dual abstract screening, full text assessments and quality assessments were only performed for a sample of the included studies. At the abstract screening stage, if there was any uncertainty about the relevance of an abstract, the abstract would be included. At the full text eligibility assessment and quality assessment stages, agreement was good and all discrepancies were minor. All outcome data were extracted independently by two reviewers. Therefore, we believe that any errors during study selection, quality assessment and data extraction are likely to be minimal and unlikely to influence the results of our review.

An additional challenge encountered was the inconsistent use of SNP nomenclature, which made gathering data for meta-analyses problematic. In particular, the *CYP2E1* SNPs considered in the primary analyses were referred to in various ways in the included studies. For example, the *CYP2E1* SNP identified by rs2031920 was referred to in articles using one or more of the following: rs2031920, “RsaI polymorphism”, “1053C > T”, “-1019C > T”, “-1055C > T”. Since rs numbers are unique to each SNP, in the first instance we identified studies reporting data for the same SNPs by using the rs numbers. If an article did not report the rs number, then we searched the literature to match the reported SNP (whatever nomenclature was used) to the rs number for that SNP. This process was especially challenging, as we were unable to identify a comprehensive database listing all the various alternative names for each SNP identified by a unique rs number.

The inconsistency in definition of hepatotoxicity across the included articles (22 different definitions across 30 articles) introduced heterogeneity into the meta-analyses. Jorgensen et al. [62] and Contopoulos-Ioannidis et al. [63] also observed variability in definitions of outcomes across pharmacogenetics studies. If outcome definitions were more comparable between pharmacogenetic studies, the extent of heterogeneity observed in meta-analyses would be reduced. In the area of TB research and clinical practice, there appears to be inconsistency in how ATDH is defined. It would be beneficial for consensus to be reached between experts in this clinical area on the definitions of outcomes that are commonly reported in pharmacogenetic studies of anti-TB drugs.

Furthermore, most studies reported that patients were treated with a combination of anti-TB drugs, meaning that it is very difficult to link pharmacogenomic factors to specific medications with the available data. It is possible that some of these studies included patients with rifampicin- or pyrazinamide-induced hepatotoxicity, for which biological mechanisms are unknown [9]. If genetic variants of the *CYP2E1* gene do not contribute to rifampicin- or pyrazinamide-induced hepatotoxicity, the inclusion of patients with rifampicin- or pyrazinamide-induced hepatotoxicity may have contributed to the lack of association identified between the *Dra*I and *Pst*I polymorphisms and ATDH.

Finally, our review is limited by the lack of evidence from studies conducted in Africa. Genotype frequencies of *CYP* genes vary greatly across the African continent [64], where TB is endemic. Only two studies included in this review were conducted in Africa; one was conducted in Tunisia [37], and one in Ethiopia [58]. Therefore, most of the evidence included in this review is not representative of the global population most affected by TB. To better understand the relationship between *CYP* genetic variants and anti-TB drug-related toxicity outcomes in African populations, more pharmacogenetic studies are required from this setting.

## Conclusions

Generally, we identified that coverage of the association between SNPs of *CYP* genes and anti-tuberculosis drug-related toxicity outcomes is incomplete. We observed significant associations between the *Rsa*I and 96-bp deletion-insertion SNPs of the *CYP2E1* gene and anti-tuberculosis drug-related hepatotoxicity. We are unaware of the publication of any other meta-analyses for SNPs of *CYP* genetic variants other than the *Rsa*I, *Dra*I and *Pst*I polymorphisms, so our results add to the existing understanding of the association between *CYP* genetic variants and hepatotoxicity. A stratified medicine approach to TB treatment would allow the benefit-risk ratio to be improved, therefore improving patient outcome and reducing healthcare costs. Whilst the findings from our meta-analyses alone lack the strength of evidence required to support a stratified approach at this time, they suggest, particularly in the case of the *CYP2E1* gene, that comprehensive genotyping in a wider range of populations is required to establish the value of pharmacogenetics testing in the treatment of TB.

## Additional files

Additional file 1: PRISMA 2009 Checklist. (DOC 53 kb)

Additional file 2: Search history. (DOCX 22 kb)

Additional file 3: **Table S1.** Key characteristics of included studies. (DOCX 40 kb)

Additional file 4: **Table S2.** Definitions of hepatotoxicity in the included studies. (DOCX 23 kb)

Additional file 5: **Table S3.** Definitions of other toxicity outcomes. (DOCX 15 kb)

Additional file 6: Results of the sensitivity analyses. (DOCX 62 kb)

Additional file 7: Funnel plots for the primary analyses. (DOCX 20 kb)

Additional file 8: *CY*P genetic variants and hepatotoxicity meta-analyses. (DOCX 38 kb)

Additional file 9: **Table S4.** Summary of results for other toxicity outcomes. (DOCX 16 kb)

## Abbreviations

ATD: Anti-tuberculosis drug
ATDH: Anti-tuberculosis drug-induced hepatotoxicity
CI: Confidence interval
CYP: Cytochrome P450
GI: Group identifier
HWE: Hardy–Weinberg equilibrium
MPE: Maculopapular eruption
OR: Odds ratio
RCT: Randomised controlled trial
SNP: Single-nucleotide polymorphism
TB: Tuberculosis
