# Supplementary material for: CYP genetic variants and toxicity related to anti-tubercular agents: a systematic review and meta-analysis
Source: Syst Rev. 2018 Nov 20;7:204. doi: 10.1186/s13643-018-0861-z (PMC6247669; doi:10.1186/s13643-018-0861-z)
Supplement: Supplementary file 2 — Search history. (DOCX 22 kb) [file 13643_2018_861_MOESM2_ESM.docx]

# Additional File 2: Search history.

| **Databases** | **Date searched** | **No. retrieved** |
| --- | --- | --- |
| MEDLINE (Ovid) and MEDLINE In-Process (Ovid) | 03/03/2016 | 3029 |
| EMBASE (Ovid) | 03/03/2016 | 4778 |
| PubMed | 03/03/2016 | 379 |
| Web of science | 03/03/2016 | 421 |
| Biosis | 03/03/2016 | 328 |

*Search Strategies*

| **Database: Web of science and BIOSIS** |
| --- |
| \| [618](http://apps.webofknowledge.com.liverpool.idm.oclc.org/summary.do?product=UA&doc=1&qid=31&SID=Y144YhJlA9U9SPEzDH1&search_mode=CombineSearches&update_back2search_link_param=yes) \| #11 AND #9 AND #8 \| \| --- \| --- \| \| [Approximately](http://apps.webofknowledge.com.liverpool.idm.oclc.org/summary.do?product=UA&doc=1&qid=30&SID=Y144YhJlA9U9SPEzDH1&search_mode=CombineSearches&update_back2search_link_param=yes)  [1,634,627](http://apps.webofknowledge.com.liverpool.idm.oclc.org/summary.do?product=UA&doc=1&qid=30&SID=Y144YhJlA9U9SPEzDH1&search_mode=CombineSearches&update_back2search_link_param=yes) \| #10 OR #7 OR #6 OR #5 OR #4 \| \| [Approximately](http://apps.webofknowledge.com.liverpool.idm.oclc.org/summary.do?product=UA&doc=1&qid=29&SID=Y144YhJlA9U9SPEzDH1&search_mode=GeneralSearch&update_back2search_link_param=yes)  [9,565](http://apps.webofknowledge.com.liverpool.idm.oclc.org/summary.do?product=UA&doc=1&qid=29&SID=Y144YhJlA9U9SPEzDH1&search_mode=GeneralSearch&update_back2search_link_param=yes) \| **TITLE:** ((((Genetic or gene*) near/2 associat* near/2 (studies or study or analys*)))) \| \| [Approximately](http://apps.webofknowledge.com.liverpool.idm.oclc.org/summary.do?product=UA&doc=1&qid=27&SID=Y144YhJlA9U9SPEzDH1&search_mode=CombineSearches&update_back2search_link_param=yes)  [93,935](http://apps.webofknowledge.com.liverpool.idm.oclc.org/summary.do?product=UA&doc=1&qid=27&SID=Y144YhJlA9U9SPEzDH1&search_mode=CombineSearches&update_back2search_link_param=yes) \| #2 OR #1 \| \| [Approximately](http://apps.webofknowledge.com.liverpool.idm.oclc.org/summary.do?product=UA&doc=1&qid=26&SID=Y144YhJlA9U9SPEzDH1&search_mode=GeneralSearch&update_back2search_link_param=yes)  [382,014](http://apps.webofknowledge.com.liverpool.idm.oclc.org/summary.do?product=UA&doc=1&qid=26&SID=Y144YhJlA9U9SPEzDH1&search_mode=GeneralSearch&update_back2search_link_param=yes) \| **TITLE:** ((((TB or Tuberculosis* or Antitubercul*)))) \| \| [Approximately](http://apps.webofknowledge.com.liverpool.idm.oclc.org/summary.do?product=UA&doc=1&qid=25&SID=Y144YhJlA9U9SPEzDH1&search_mode=GeneralSearch&update_back2search_link_param=yes)  [219,324](http://apps.webofknowledge.com.liverpool.idm.oclc.org/summary.do?product=UA&doc=1&qid=25&SID=Y144YhJlA9U9SPEzDH1&search_mode=GeneralSearch&update_back2search_link_param=yes) \| **TITLE:** ((((gene* or genetic*) near/5 (mutat* or variant*)))) \| \| [Approximately](http://apps.webofknowledge.com.liverpool.idm.oclc.org/summary.do?product=UA&doc=1&qid=24&SID=Y144YhJlA9U9SPEzDH1&search_mode=GeneralSearch&update_back2search_link_param=yes)  [1,388,831](http://apps.webofknowledge.com.liverpool.idm.oclc.org/summary.do?product=UA&doc=1&qid=24&SID=Y144YhJlA9U9SPEzDH1&search_mode=GeneralSearch&update_back2search_link_param=yes) \| **TITLE:** ((((SNP or Genotyp* or Phenotyp* or Allele* or Pharmacogenet* or Pharmacogenom* or Polymorph*)))) \| \| [Approximately](http://apps.webofknowledge.com.liverpool.idm.oclc.org/summary.do?product=UA&doc=1&qid=23&SID=Y144YhJlA9U9SPEzDH1&search_mode=GeneralSearch&update_back2search_link_param=yes)  [38,430](http://apps.webofknowledge.com.liverpool.idm.oclc.org/summary.do?product=UA&doc=1&qid=23&SID=Y144YhJlA9U9SPEzDH1&search_mode=GeneralSearch&update_back2search_link_param=yes) \| **TITLE:** (((single* near/2 nucleotid* near/2 polymorph*))) \| \| [Approximately](http://apps.webofknowledge.com.liverpool.idm.oclc.org/summary.do?product=UA&doc=1&qid=22&SID=Y144YhJlA9U9SPEzDH1&search_mode=GeneralSearch&update_back2search_link_param=yes)  [45,745](http://apps.webofknowledge.com.liverpool.idm.oclc.org/summary.do?product=UA&doc=1&qid=22&SID=Y144YhJlA9U9SPEzDH1&search_mode=GeneralSearch&update_back2search_link_param=yes) \| **TITLE:** ((((genetic* or gene*) near/3 (suscept* or predisposit* or anticipat*)))) \| \| [Approximately](http://apps.webofknowledge.com.liverpool.idm.oclc.org/summary.do?product=UA&doc=1&qid=20&SID=Y144YhJlA9U9SPEzDH1&search_mode=GeneralSearch&update_back2search_link_param=yes)  [47,961](http://apps.webofknowledge.com.liverpool.idm.oclc.org/summary.do?product=UA&doc=1&qid=20&SID=Y144YhJlA9U9SPEzDH1&search_mode=GeneralSearch&update_back2search_link_param=yes) \| **TITLE:** (((aminosalicylic acid or diarylquinoline* or ethambutol* or ethionamide* or isoniazid* or prothionamide* or pyrazinamide* or thioacetazone* or capreomycin* or cycloserine* or enviomycin* or rifabutin* or rifampin* or viomycin*))) \| \| [Approximately](http://apps.webofknowledge.com.liverpool.idm.oclc.org/summary.do?product=UA&doc=1&qid=19&SID=Y144YhJlA9U9SPEzDH1&search_mode=GeneralSearch&update_back2search_link_param=yes)  [49,386](http://apps.webofknowledge.com.liverpool.idm.oclc.org/summary.do?product=UA&doc=1&qid=19&SID=Y144YhJlA9U9SPEzDH1&search_mode=GeneralSearch&update_back2search_link_param=yes) \| **TITLE:** ((((Antitubercul* or tuberculos* or TB) Near/4 (agent* or drug* or antibiotic* or medicine* or medication* or treatment*)))) \| |

| **Database: MEDLINE** |
| --- |
| \| [# ▲](http://ovidsp.tx.ovid.com/sp-3.18.0b/ovidweb.cgi?&S=OPDEFPAOGIDDHCAFNCJKEHFBGBANAA00&Sort+Sets=descending) \| **Searches** \| **Results** \| \| --- \| --- \| --- \| \| 1 \| antitubercular agents/ or aminosalicylic acid/ or diarylquinolines/ or ethambutol/ or ethionamide/ or isoniazid/ or prothionamide/ or pyrazinamide/ or thioacetazone/ or antibiotics, antitubercular/ or capreomycin/ or cycloserine/ or enviomycin/ or rifabutin/ or rifampin/ or viomycin/ \| 73943 \| \| 2 \| ((Antitubercul* or tuberculos* or TB) adj4 (agent* or drug* or antibiotic* or medicine* or medication* or treatment*)).tw. \| 29293 \| \| 3 \| (aminosalicylic acid or diarylquinoline* or ethambutol* or ethionamide* or isoniazid* or prothionamide* or pyrazinamide* or thioacetazone* or capreomycin* or cycloserine* or enviomycin* or rifabutin* or rifampin* or viomycin*).tw. \| 26053 \| \| 4 \| 1 or 2 or 3 \| 93357 \| \| 5 \| Polymorphism, Genetic/ \| 103705 \| \| 6 \| genetic predisposition to disease/ or anticipation, genetic/ \| 101390 \| \| 7 \| Pharmacogenetics/ \| 9595 \| \| 8 \| Genetic Association Studies/ \| 14210 \| \| 9 \| ((Genetic or gene*) adj2 associat* adj2 (studies or study or analys*)).tw. \| 4883 \| \| 10 \| ((genetic* or gene*) adj3 (suscept* or predisposit* or anticipat*)).tw. \| 40247 \| \| 11 \| Polymorphism, Single Nucleotide/ \| 77811 \| \| 12 \| (single* adj2 nucleotid* adj2 polymorph*).tw. \| 46260 \| \| 13 \| (SNP or Genotyp* or Phenotyp* or Allele* or Pharmacogenet* or Pharmacogenom* or Polymorph*).tw. \| 774469 \| \| 14 \| ((gene* or genetic*) adj5 (mutat* or variant*)).tw. \| 182197 \| \| 15 \| Genotype/ or Phenotype/ or Alleles/ \| 381555 \| \| 16 \| or/5-15 \| 1035512 \| \| 17 \| exp Tuberculosis/ \| 175110 \| \| 18 \| (TB or Tuberculosis*).tw. \| 153175 \| \| 19 \| Antitubercul*.tw. \| 11635 \| \| 20 \| or/17-19 \| 213138 \| \| 21 \| 4 and 16 and 20 \| 2846 \| \| 22 \| animal/ not human/ \| 4159388 \| \| **23** \| **21 not 22** \| **2730** \| |

| **Database: EMBASE** |
| --- |
| \| \| [# ▲](http://ovidsp.uk.ovid.com/sp-3.25.0a/ovidweb.cgi?&S=POIHPDPCNBHFIMKMFNGKPFDGBNGJAA00&Sort+Sets=descending) \| Searches \| Results \| \| --- \| --- \| --- \| \| 1 \| antitubercular agents/ or aminosalicylic acid/ or diarylquinolines/ or ethambutol/ or ethionamide/ or isoniazid/ or prothionamide/ or pyrazinamide/ or thioacetazone/ or antibiotics, antitubercular/ or capreomycin/ or cycloserine/ or enviomycin/ or rifabutin/ or rifampin/ or viomycin/ \| 151901 \| \| 2 \| ((Antitubercul* or tuberculos* or TB) adj4 (agent* or drug* or antibiotic* or medicine* or medication* or treatment*)).tw. \| 40664 \| \| 3 \| (aminosalicylic acid or diarylquinoline* or ethambutol* or ethionamide* or isoniazid* or prothionamide* or pyrazinamide* or thioacetazone* or capreomycin* or cycloserine* or enviomycin* or rifabutin* or rifampin* or viomycin*).tw. \| 34743 \| \| 4 \| 1 or 2 or 3 \| 172588 \| \| 5 \| Polymorphism, Genetic/ \| 102257 \| \| 6 \| genetic predisposition to disease/ or anticipation, genetic/ \| 97585 \| \| 7 \| Pharmacogenetics/ \| 17431 \| \| 8 \| Genetic Association Studies/ \| 876 \| \| 9 \| ((Genetic or gene*) adj2 associat* adj2 (studies or study or analys*)).tw. \| 7890 \| \| 10 \| ((genetic* or gene*) adj3 (suscept* or predisposit* or anticipat*)).tw. \| 61544 \| \| 11 \| Polymorphism, Single Nucleotide/ \| 98303 \| \| 12 \| (single* adj2 nucleotid* adj2 polymorph*).tw. \| 75841 \| \| 13 \| (SNP or Genotyp* or Phenotyp* or Allele* or Pharmacogenet* or Pharmacogenom* or Polymorph*).tw. \| 1171894 \| \| 14 \| ((gene* or genetic*) adj5 (mutat* or variant*)).tw. \| 294715 \| \| 15 \| Genotype/ or Phenotype/ or Alleles/ \| 777386 \| \| 16 \| or/5-15 \| 1548879 \| \| 17 \| exp Tuberculosis/ \| 197008 \| \| 18 \| (TB or Tuberculosis*).tw. \| 187590 \| \| 19 \| Antitubercul*.tw. \| 16330 \| \| 20 \| or/17-19 \| 253048 \| \| 21 \| 4 and 16 and 20 \| 5380 \| \| 22 \| animal/ not human/ \| 1357016 \| \| 23 \| 21 not 22 \| 5360 \| \| 24 \| limit 23 to em=188300-201608 \| 4778 \| \| \| --- \| --- \| --- \| --- \| --- \| --- \| --- \| --- \| --- \| --- \| --- \| --- \| --- \| --- \| --- \| --- \| --- \| --- \| --- \| --- \| --- \| --- \| --- \| --- \| --- \| --- \| --- \| --- \| --- \| --- \| --- \| --- \| --- \| --- \| --- \| --- \| --- \| --- \| --- \| --- \| --- \| --- \| --- \| --- \| --- \| --- \| --- \| --- \| --- \| --- \| --- \| --- \| --- \| --- \| --- \| --- \| --- \| --- \| --- \| --- \| --- \| --- \| --- \| --- \| --- \| --- \| --- \| --- \| --- \| --- \| --- \| --- \| --- \| --- \| --- \| --- \| |

| **Database: PubMed** |
| --- |
| \| #1 \| Search (((Antitubercul* or tuberculos* or TB))) AND ((agent* or drug* or antibiotic* or medicine* or medication* or treatment*)) \| 124242 \| \| --- \| --- \| --- \| \| #2 \| Search ((aminosalicylic acid or diarylquinoline* or ethambutol* or ethionamide* or isoniazid* or prothionamide* or pyrazinamide* or thioacetazone* or capreomycin* or cycloserine* or enviomycin* or rifabutin* or rifampin* or viomycin*)) \| 49591 \| \| #3 \| Search (#1 or #2) \| 151329 \| \| #4 \| Search ((((Genetic or gene*) near/2 near/2 ))) AND associat*) AND ((studies or study or analys*)) \| 3922 \| \| #5 \| Search (((genetic* or gene*))) AND ((suscept* or predisposit* or anticipat*)) \| 235548 \| \| #6 \| Search ((single*) AND nucleotid*) AND polymorph* \| 98071 \| \| #7 \| Search ((SNP or Genotyp* or Phenotyp* or Allele* or Pharmacogenet* or Pharmacogenom* or Polymorph*)) \| 997538 \| \| #8 \| Search (((gene* or genetic*))) AND ((mutat* or variant*)) \| 743819 \| \| #9 \| Search (#4 or #5 or #6 or #7 or #8) \| 1553428 \| \| #10 \| Search (((((TB or Tuberculosis* or Antitubercul*))))) \| 251923 \| \| #11 \| Search (#3 and #9 and #10) \| 7671 \| \| #12 \| Search ("2015/08/01"[Date - Entrez] : "3000"[Date - Entrez]) \| 658085 \| \| #13 \| Search (#11 and #12) \| 379 \| |
