# Supplementary material for: CYP genetic variants and toxicity related to anti-tubercular agents: a systematic review and meta-analysis
Source: Syst Rev. 2018 Nov 20;7:204. doi: 10.1186/s13643-018-0861-z (PMC6247669; doi:10.1186/s13643-018-0861-z)
Supplement: Supplementary file 3 — Table S1. Key characteristics of included studies. (DOCX 40 kb) [file 13643_2018_861_MOESM3_ESM.docx]

**Additional file 3: Table S1. Key characteristics of included studies.**

| **Study** | **Country** | **Study design** | **Follow-up time** | **Drugs and dosage** | **Selection criteria** | **Sample size** | **Toxicity outcomes** |
| --- | --- | --- | --- | --- | --- | --- | --- |
| **An 2012** | China | Case-control | 6 months | Daily treatment with INH, RIF, PZA and EMB for 2 months, followed by 4 months treatment with INH and RIF, with drug dosages calculated according to body weight  Body weight < 45 kg: RIF 300 mg, INH 200 mg, PZA 1000 mg  Body weight of 45–55 kg: RIF 450 mg, INH 300 mg, PZA 1500 mg  Body weight > 55 kg: RIF 600 mg, INH 400 mg, PZA 2000 mg | Inclusion criteria:   - Daily treatment with INH, RIF, PZA and EMB for 2 months, followed by 4 months treatment with INH and RIF, with drug dosages calculated according to bodyweight - normal serum ALT, AST and bilirubin levels, no symptoms related to abnormal liver function (i.e. jaundice) prior to anti-TB drug treatment and close monitoring of changes in liver function within 6 months of treatment - patients with and without hepatotoxicity during drug treatment   Exclusion criteria:   - malnutrition - HIV type 1 infection - alcoholic liver disease or habitual drinking - hepatitis B or C infection, liver disease, systemic diseases and/or treatment with drugs other than the anti-TB drugs that can induce hepatotoxicity - severe TB or cardiac dysfunction that may cause liver dysfunction   transient increases in ALT | 208 | Hepatotoxicity |
| **Bose 2011** | India | Prospective cohort | Patients were followed up to 8 weeks after they had started with ATT | All patients received ATT (RIF, INH, EMB, and PZA) according to their body weight. All 4 drugs were given for 2 months. PZA and EMB were discontinued, while INH and RIF were continued for another 4 months  RIF, body weight (kg) and mg/day <=35: 300  36–50: 450 >50: 600  INH, body weight (kg) and mg/day <=35: 200 >35: 400  PZA, body weight (kg) and g/day <=50: 1.0 >50: 1.5  EMB 20 mg/kg/day | Newly diagnosed patients of pulmonary TB. The baseline LFT of the patients was normal when they were started on ATT  Exclusion criteria: no history of habitual alcoholism, chronic liver diseases, or steatosis | 218 | ATT induced hepatitis  Adverse DIH outcome |
| **Brito 2014** | Brazil | Prospective Cohort | NR | Daily treatment with INH, RIF, and PZA for the first 2 months, followed by INH and RIF for an additional 4 months | Inclusion criteria: patients aged 18 years and older that were newly diagnosed with active TB and were submitted to treatment as described in “Drugs and dosage”.  Exclusion criteria: patients using anti-TB drugs prior to study enrollment, patients whose results of LFTs prior to the beginning of treatment were higher than twice the ULN and refusal to participate in the study. | 245 | Drug-induced hepatitis |
| **Chamorro 2013** | Argentina | Prospective cohort | NR | The patients began a standard TB-treatment protocol for the first 2 months (INH: 5 mg/kg/days, maximum 300 mg/day; RIF: 10 mg/kg/day, maximum 600 mg/day; PZA: 20 mg/kg/day; EMB: 20 mg/kg/day), followed by INH and RIF for 4 months or more, depending on the disease severity or the presence of extrapulmonary foci. | Inclusion criteria: TB patients who were over age 18 and had stable hemodynamic levels, normal renal function, and had tested negative for pregnancy  Exclusion criteria: presence of diseases that directly affected the liver (acute hepatitis A, active B, and C; cirrhosis; encephalopathy, and cancer), INH allergy, HIV, autoimmunity, concomitant hepatotoxic medications, a history of TB-treatment failures, refusal of blood extraction, and refusal to sign the written informed consent. | 175 | ATDH |
| **Cho 2007** | Korea | Prospective cohort | Serum AST, ALT, and total bilirubin levels were then monitored monthly until the end of treatment | All patients received oral INH (300 mg), RIF (600 mg), PZA (20 mg/kg body weight), and EMB (800 mg) daily for the first 2 months. PZA was then discontinued, while INH, RIF and EMB were continued for another 4 months. | Adult patients newly diagnosed with active TB, having evident lesion of TB by simple X-ray or computed tomography or positive results of sputum smear or culture for detection of mycobacteria  Exclusion criteria: (1) abnormal serum ALT, AST, or bilirubin levels or symptoms related to abnormal liver function such as jaundice before anti-TB treatment; (2) alcoholic liver disease or habitual alcohol drinking; (3) any other hepatic or systemic diseases that may cause liver dysfunction. | 132 | ATDH |
| **Costa 2012** | Brazil | Prospective cohort | NR | All patients were treated with the first-line anti-TB drug regimen INH (300 mg/kg/day), RIF (300 mg/kg/day), and PZA (1500 mg/kg/day) for the first 2 months, and then INH and RIF for a further 4 months. | Male or female subjects aged 18 years or over, who had no previously described renal, allergic, or hepatic diseases and were not pregnant, were considered for the study. | 129 | ADRs |
| **Feng 2014** | China | Case-control | 6 months | Treatment with anti-TB drug regimens at the usual dosage, including 300 mg/day INH, 450 mg/day RIF, and 1500 mg/day PZA | Selection of cases:  The cases were selected based on liver functions, i.e., all indices of liver function were normal before anti-TB chemotherapy, and became abnormal indicating hepatic injury after 6 months of chemotherapy. Cases were patients who showed anti-TB drug-induced hepatitis based on increased serum transaminase values that were 3-fold higher than the ULN (40 IU/L ALT) and symptoms compatible with hepatitis.  Selection of controls: Controls underwent the same anti-TB chemotherapy with the selected cases and were not tested with abnormality in liver functions after 6 months of the chemotherapy. The controls selected matched the criteria compared to the cases: i) same gender; ii) age discrepancy of less than 5 years; iii) living in the same regions; and iv) treatment with anti-TB drug regimens at the usual dosage, including 300 mg/day INH, 450 mg/day RIF, and 1500 mg/day PZA. | 346 | ATDH |
| **Fernandes 2015 (GI: SANTOS)** | Brazil | Prospective Cohort | NR | INH, RIF, and PZA for the first 2 months, followed by INH and RIF daily for 4 months. | Inclusion criteria: all had been diagnosed with TB and treated as described in “Drugs and Dosage”.    Exclusion criteria: age <18 years, mental disability, chronic liver disease confirmed by clinical and laboratory data, anti-TB drug use prior to enrolment in the study, and LFT results before beginning treatment that were greater than twice the ULN. | 220 | DIH |
| **Fredj 2016** | Tunisia | Prospective cohort | Serum AST, ALT and ALP were monitored monthly until the end of the treatment | INH (5 mg kg− 1 per day), RIF (10 mg kg− 1 per day), PZA (25 mg kg− 1 per day) and EMB (15 mg kg− 1 per day) for the first 2 months, followed by INH and RIF during 4 to 7 additional months, depending on TB clinical presentation | Patients diagnosed with pulmonary and extra-pulmonary TB | 71 | IIH |
| Gogtay 2016 | India | Case-control | NR | Anti-tuberculous medications - no further details provided | Cases: patients receiving anti-tuberculous medications; patients with symptoms/signs such as anorexia, nausea, vomiting, malaise, icterus, and raised serum aminotransferase levels greater than 2 times the ULN value or greater than 5 times the ULN without clinical symptoms were considered to have hepatotoxicity. Only those who had never consumed alcohol and whose hepatitis B serology was negative were included as cases.   Controls: those on anti-tuberculous medications who had never had any clinical symptoms or abnormal LFTs suggestive of hepatotoxicity.  Healthy population: Inclusion criteria were healthy, unrelated adults (>18 years) of either gender adjudged normal by history, physical examination, and laboratory investigations including HIV and hepatitis B surface antigen serology and who were either non-smokers or had refrained from smoking for the past 3 months.  Exclusion criteria were chronic alcoholics, alcohol or caffeine consumption in the past 24 h or paracetamol consumption in the past 7 days. | Cases: 25 Controls: 50  Healthy population: 139 | ATDH |
| **Gupta 2013**  **(GI: GUPTA)** | India | Prospective cohort | The patients were monitored for ALT, AST, and total bilirubin levels weekly for 1 month and then monthly until the completion of treatment | Initially patients received a combination regimen including INH 5 mg/Kg (maximum 300 mg daily), RIF 10 mg/Kg (maximum 600 mg daily), PZA 25 mg/Kg (maximum 1500 mg daily), and EMB 15–25 mg/Kg (maximum 2000 mg daily) for a period of 2 months and then for an additional 4 months with INH and RIF. | Inclusion criteria were: i) age above 18 years; (ii) positive smear and/or culture for detection of mycobacteria in clinical samples; and (iii) normal ALT, AST and total bilirubin levels.  Exclusion criteria were (i) patients presenting clinically and laboratory confirmed chronic liver disease such as jaundice; (ii) acute and chronic hepatitis B and/or C or HIV; (iii) alcoholic liver diseases; (iv) a rise of 2 times the ULN of ALT, AST and total bilirubin levels; (v) medication with anti-TB drugs prior to beginning of the treatment and/or other potentially hepatotoxic drugs; and (vi) refusal to provide blood sample or signed informed consent form. | 215 | ATDH |
| **He 2015** | China | Case-control | 6 months | Daily 2S(E)HRZ4HR: S, streptomycin; E, ethambutol; H, isoniazid, R, rifampicin; Z, pyrazinamide; dose increased for 2 months and then consolidated for 4 months | Cases:  Inclusion criteria: occurrence of liver injury after 6 months of anti-TB drug therapy.  Controls:  Inclusion criteria: absence of liver injury after 6 months of anti-TB drug therapy and a match with patients in the case group in terms of age (<5 years difference), sex, and therapeutic regimen  Exclusion criteria: presence of abnormal liver function before the administration of TB treatment; co-occurrence of other diseases that can cause liver function abnormalities such as viral hepatitis, alcoholic liver disease, autoimmune hepatitis, and hypoxemia; and consumption of other drugs that can cause liver dysfunction in patients. | 254 | ADLI |
| **Huang 2003** | Taiwan | Prospective cohort | Serum ALT, AST, and total bilirubin levels were monitored monthly until the end of treatment or checked whenever patients had symptoms of suspected hepatitis | Their standard daily anti-TB regimen for the first 2 months included INH (300 mg), RIF (600 mg or 450 mg if body weight <50 kg), PZA (20 mg/kg body weight), and EMB (25 mg/kg body weight). PZA was then discontinued, whereas INH, RIF, and EMB (15 mg/kg body weight) were continued for another 4 months | Patients with incident pulmonary or extrapulmonary TB  Exclusion criteria were: i) abnormal serum ALT, AST, or bilirubin before anti-TB treatment; and ii) refusal of blood sampling or informed written consent | 318 | ADIH |
| **Kim 2009 (GI: KIM)** | Korea | Case-control | Assessments performed 2 weeks after onset of treatment and bi-monthly thereafter | All patients with pulmonary TB were treated daily with a combination regimen including INH (300-400 mg daily), RFP (450-600 mg daily), EMB (600-800 mg daily) and PZA (1000-1500 mg daily) for 2 months and then without PZA for 4 or more following months. Doses of each drug were adjusted based on body weight of the patient | Newly diagnosed and treated patients with pulmonary TB.  Exclusion criteria: patients with active or chronic hepatitis including alcoholic hepatitis, fatty liver disease, liver cirrhosis, carriers of the hepatitis B or C virus, heavy alcohol intake, decreased renal function and severe cardiac diseases requiring several medications | 226 | ATDH |
| **Kim 2011 (GI: KIM)** | Korea | Case-control | NR | The treatment consisted of an initial phase of 2 months and a subsequent continuation phase of 4 or more months. During the initial phase, 4 drugs were administered including INH (300–400 mg daily), RIF (450–600 mg daily), EMB (600–800 mg daily), and PZA (1,000–1,500 mg daily). Doses of each drug were adjusted based on the body weight of the patient. In the following continuation phase, only PZA was discontinued, while the other 3 drugs were continued. | Patients newly diagnosed with pulmonary TB and/or TB pleuritis and treated with first-line anti-TB medications such as INH, RIF, EMB, and PZA  Exclusion criteria:   - Patients with skin diseases before treatment - Chronic renal failure and chronic liver diseases affecting drug metabolism - Chronic alcoholism - Other chronic medical conditions requiring medication   Non-adherence to the treatment | 221 | ATD-induced MPE |
| **Lee 2010** | Taiwan | Prospective cohort | NR | All patients received oral INH 300 mg, RIF 600 mg (or 450 mg if body weight was <50 kg), PZA 200 mg/kg body weight and EMB 800 mg daily for the first 2 months. PZA was then discontinued, while INH, RIF and EMB were continued for another 4 months | Inclusion criteria: adult patients newly diagnosed with active TB, having evident lesions of TB by simple X-ray, computed tomography, positive results of sputum smears and cultures for detection of mycobacteria  Exclusion criteria: 1) positive serum hepatitis B virus surface antigen, antibody to hepatitis C virus; 2) alcoholic liver disease or habitual alcohol drinking; 3) any other hepatic or systemic diseases that may cause liver dysfunction; 4) abnormal serum ALT, AST or bilirubin levels before anti-TB treatment | 140 | ATDH |
| **Rana 2014** | India | Prospective cohort | Patients were monitored every month till the end of treatment or whenever the patients had symptoms or signs of hepatotoxicity | Daily ATT for the first 2 months included INH (300 mg), RIF (600 or 450 mg for body weight/50 kg), PZA (20 mg/kg body weight) and EMB (25 mg/kg body weight). After 2 months, EMB and PZA were discontinued, whereas INH and RIF were continued for an additional 4 months | Inclusion criteria: Patients with pulmonary and extra-pulmonary TB  Exclusion criteria: patients with history of alcohol abuse and/or any other liver disease; patients who had received other potentially hepatotoxic drugs in addition to antitubercular drugs; patients who had abnormal serum ALT, AST or bilirubin before starting ATT; patients who developed viral hepatitis during ATT; patients with renal failure or cancer; and patients who refused blood sampling or providing informed written consent | 300 | Hepatotoxicity |
| **Roy 2006** | India | Prospective Cohort | NR | Most of the patients were treated with INH (5 mg/kg bodyweight per day); RIF (10 mg/kg bodyweight per day) and PZA (20–35 mg/kg bodyweight per day). A few patients with TB meningitis also received EMB (20 mg/kg bodyweight per day). After 2 months, PZA and EMB were discontinued and INH and RIF were continued for the next 4 months | Paediatric patients with TB – no further information provided | 109 | ATDH |
| **Santos 2013 (GI: SANTOS)** | Brazil | Prospective cohort | NR | Treatment with INH, RIF and PZA for the first 2 months, followed by INH and RIF daily for 4 months. | Inclusion criteria: patients diagnosed with TB and treated with anti-TB treatment (see drugs and dosage)  Exclusion criteria: patients aged <18 years, those with mental disabilities, chronic liver disease confirmed by clinical and laboratory data, users of anti-TB drugs before enrolment in the study, and those with liver function results higher than 2 times the ULN before beginning treatment. | 270 | ATDH |
| **Sharma 2014** | India | Case-control | NR | INH, RIF, PZA, EMB; dosages administered to patients according to body weight:  RIF, body weight (kg) and mg/day <=35: 300  36–50: 450 >50: 600  INH, body weight (kg) and mg/day <=35: 200 >35: 300  PZA, body weight (kg) and g/day <=50: 1.0 >50: 1.5  EMB, 15 mg/kg/day | Cases: patients who developed clinical and/or laboratory evidence of DIH while on ATT  Controls: TB patients without DIH  Exclusion criteria: patients whose serum samples tested positive for markers of viral hepatitis and/or who were receiving other potentially hepatotoxic drugs or had ultrasonography evidence of chronic liver disease; HIV-infected and chronic alcohol-dependent patients who had consumed >48 g of alcohol/day for at least 1 year; patients receiving other potentially hepatotoxic drugs (e.g., methotrexate, phenytoin, valproate, fluconazole); pregnant women; subjects who did not provide written informed consent | 314 | DIH |
| **Singla 2014** | India | Prospective cohort | NR | NR | Inclusion criteria: newly diagnosed patients with TB  Exclusion criteria: history of heavy use of alcohol or chronic liver diseases or liver cirrhosis; patients who were settled in the area for a minimum of 3 generations; people infected with HIV | 408 | ATDH |
| **Sotsuka 2011** | Japan | Prospective cohort | 3 months | INH, RIF and PZA, plus EMB or SM during the first 2 months, followed by administration of INH and RIF plus EMB or SM during the final 4 months | Inclusion criteria: inpatients with active pulmonary TB who were treated with the standard Japanese chemotherapy regimen followed up for more than 3 months after treatment, and who consented to this study | 144 | Hepatotoxicity |
| **Tang 2012 (GI: ADACS)** | China | Case-control | Patients were monitored for 6–9 months according to the treatment episode. | All patients took INH 600 mg, RIF 600 mg (or 450 mg if body weight was < 50 kg), PZA 2000 mg and EMB 1250 mg every other day for the first 2 months (re-treatment patients were injected with SM 750 mg each time simultaneously). PZA and EMB were then discontinued for primary patients, whereas INH and RIF were continued for another 4 months. PZA and SM were discontinued for re-treatment patients, whereas INH, RIF and EMB continued. | Inclusion criteria: sputum smear-positive patients who received standard short-course chemotherapy recommended by WHO  Exclusion criteria: patients using other potentially hepatotoxic medications; patients with positive serum hepatitis B virus surface antigen, alcohol drinking, liver diseases or abnormal liver function before ATT | 445 | ATDH |
| **Tang 2013a (GI: ADACS)** | China | Case-control | Patients were monitored for 6-9 months according to the treatment episode | All patients took INH 600 mg, RIF 600 mg (or 450 mg if body weight was < 50 kg), PZA 2000 mg and EMB 1250 mg every other day for the first 2 months (re-treatment patients were injected with SM 750 mg each time simultaneously). PZA and EMB were then discontinued for primary patients, while INH and RIF were continued for another 4 months. PZA and SM were discontinued for re-treatment patients, while INH, RIF and EMB continued. | Inclusion criteria: newly diagnosed patients with sputum smear positive pulmonary TB  Exclusion criteria: 1) abnormal serum ALT, AST or total bilirubin levels before ATT; 2) positive serum hepatitis B virus surface antigen; 3) alcoholic liver disease or habitual alcohol drinking; 4) concomitant use of hepatotoxic drugs; 5) a history of chronic liver disease or systemic diseases that may cause liver dysfunction | 445 | ATDH |
| **Tang 2013b (GI: ADACS)** | China | Case-control | NR | All patients received standard ATT for 6–9 months. Patients were given 600 mg INH, 600 mg RIF (or 450 mg if bodyweight was <50 kg), 2000 mg PZA and 1250 mg EMB every other day in the first 2 months (patients undergoing retreatment were injected i.m. simultaneously with 750 mg SM each time). Then, PZA and EMB were discontinued in patients undergoing treatment for the first time, whereas INH and RIF were continued for another 4 months. In patients undergoing retreatment, PZA and SM were discontinued after the first 2 months, whereas INH, RIF and EMB were continued for another 4 months | Inclusion criteria: newly diagnosed patients with sputum smear-positive pulmonary TB  Exclusion criteria: (i) abnormal serum ALT, AST or total bilirubin levels  before ATT; (ii) positive serum hepatitis B virus surface antigen; (iii) alcoholic liver disease or habitual alcohol drinking (defined as drinking a glass of wine or a can of beer at least once a week for a period of > 3 months); (iv) the concomitant use of hepatotoxic drugs, including Chinese herbal medicines, antibiotics (cephems, quinolone, drugs for anaerobes, erythromycin, amoxicillin), drugs for the treatment of hyperlipidaemia and drugs for the treatment of hyperthyroidism; and (v) a history of chronic liver disease or systemic diseases that may cause liver dysfunction | 445 | ATDH |
| **Teixeira 2011** | Brazil | Case-control | NR | Anti-TB drug regimens that include INH at the usual dosage (400 mg/day) | Inclusion criteria: (i) age above 18 years, (ii) diagnosis of active TB, (iii) treatment with anti-TB drug regimens that include INH at the usual dosage (400 mg/day) and (iv) normal baseline serum transaminases (ALT and AST) before treatment  Exclusion criteria: i) positive serological test for the HIV, hepatitis B virus or hepatitis C virus, (ii) alcohol abuse, (iii) history of chronic liver disease and (iv) pregnancy | 167 | Hepatitis |
| **Wang 2010** | China | Case-control | NR | INH, RIF, PZA, and EMB for 2 months followed by INH and RIF for 4 months (2HRZE/4HR) | TB patients with (cases) and without (controls) ADIH  Exclusion criteria: patients with chronic hepatitis B virus infection, alcohol consumption, obesity, senility and poor nutritional status | 215 | ADIH |
| **Wang 2011** | Taiwan | Prospective cohort | LFT was checked at 2, 4, 6, 8, 12, and 16 weeks after the start of anti-TB treatment, or whenever symptoms of hepatitis developed and clinically relevant hepatitis was suspected by the primary care physician during the course of treatment | All participants received a standard anti-TB treatment of daily INH, RIF, EMB, and PZA in the first 2 months, and daily INH and RIF for the succeeding 4 months.  The regimen was modified if necessary by the primary care physician. | Inclusion criteria: adult Taiwanese (>16 years) with culture-confirmed pulmonary TB  Exclusion criteria: patients were excluded if they were pregnant, had a life expectancy <6 months, had abnormal baseline LFT due to congestive heart failure, hepatic malignancy or alcohol abuse, or had Mycobacterium tuberculosis isolates resistant to either INH or RIF | 360 | Drug-induced HATT |
| **Xiang 2014** | China | Prospective cohort | 2 months | All patients were prescribed INH (600 mg), RIF (600 mg, or 450 mg if the body weight was less than 50 kg), PZA (2,000 mg), and EMB (1,250 mg) every other day in the first 2 months. After 2 months, INH and were continued for a further 4 to 6 months. Retreatment patients in addition received SM (750 mg) every other day in the first 2 months and continued receiving EMB for another 6 months | Inclusion criteria: newly diagnosed pulmonary TB patients belonging to the Uyghur ethnic group, who were receiving standard short-course chemotherapy recommended by WHO, who attended for a 2-month assessment, and any patients attending clinic with suspected liver disease after the start of treatment, prior to the 2-month visit  Exclusion criteria: patients who had signs of abnormal liver function when they started treatment (jaundice or elevated ALT, AST or bilirubin levels), or disease associated with liver dysfunction | 2244 | ATLI |
| **Yamada 2009** | Canada | Prospective cohort | 9 months | INH 300 mg | Subjects who were receiving treatment with INH 300mg daily self-administered for latent TB infection. Subjects were included if they were: aged 19 years of age or older; not concurrently receiving other anti-TB drugs; nonreactive to hepatitis B surface antigen and antibody to hepatitis C virus; absence of any liver or metabolic diseases; without a HIV-positive test result; not consuming 7 or more alcoholic beverages per day and undergoing sufficiently frequent AST tests to detect hepatotoxicity | 170 | Hepatotoxicity |
| **Yimer 2011** | Ethiopia | Prospective cohort | Followed up for development of DILI for up to 56 weeks | All study participants received RIF based short-course chemotherapy for TB following the national TB treatment guideline. ART was then initiated. | Newly diagnosed ART and anti-TB treatment naïve adult TB and HIV co-infected patients. The eligibility criteria were age >18 years, CD4 count, 200 cells/UL, not pregnant and not on other known hepatotoxic drugs concurrently (except co-trimoxazole, 960 mg per day, which was given to all participants before enrolment and during the follow-up period according to the treatment guideline). | 353 | Anti-tubercular and antiretroviral drugs induced liver injury |
| **Zaverucha-do-Valle 2014** | Brazil | Retrospective cohort | Follow-up at days 15 and 30 and at least 1 visit monthly until the end of therapy. | 600 mg/day of RIF, 400 mg/day of INH and 2 g/day of PZA for all patients with corporal weight >45 kg or adjusted for corporal weight < 45 kg. After 2 months of therapy, PZA was discontinued | Inclusion criteria: signed written consent, sputum smear with acid-fast bacilli or culture positive for Mycobacterium tuberculosis; ongoing TB treatment and laboratory tests of liver function.  Exclusion criteria: age <18 years, pregnancy and no more than 1 visit registered | 131 | ATDH |

ADIH=anti-tuberculosis drug-induced hepatitis; ALP=alkaline phosphatase; ALT=alanine aminotransferase; AST=aspartate aminotransferase; ATT=anti-tuberculosis treatment; ATD=anti-tuberculosis drug; ATDH=anti-tuberculosis drug-induced hepatotoxicity; ATLI=anti-tuberculosis drug-induced liver injury; DIH=drug-induced hepatotoxicity; DILI=drug-induced liver injury; EMB=ethambutol; HATT=hepatotoxicity during anti-tuberculosis treatment; IIH=isoniazid-induced hepatotoxicity; INH=isoniazid; LFT=liver function test; MPE=maculopapular eruption; NR=not reported; PZA=pyrazinamide; RIF=rifampicin; SM=streptomycin; TB=tuberculosis; ULN=upper limit of normal; WHO=World Health Organization
