# Supplementary material for: CYP genetic variants and toxicity related to anti-tubercular agents: a systematic review and meta-analysis
Source: Syst Rev. 2018 Nov 20;7:204. doi: 10.1186/s13643-018-0861-z (PMC6247669; doi:10.1186/s13643-018-0861-z)
Supplement: Supplementary file 4 — Table S2. Definitions of hepatotoxicity in the included studies. (DOCX 23 kb) [file 13643_2018_861_MOESM4_ESM.docx]

**Additional file 4: Table S2. Definitions of hepatotoxicity in the included studies.**

| **Study** | **Outcome and definition** |
| --- | --- |
| **An 2012** | ATDH was defined as an increase of over 2 times the ULN range in ALT or conjugated bilirubin levels or a concurrent increase in AST levels, according to the criteria of drug-induced liver injury developed at an international consensus meeting [1]. |
| **Bose 2011, Yimer 2011** | ATDH (Bose 2011)/DILI (Yimer 2011) in patients was defined according to the international consensus criteria [1]. Liver biochemical parameters more than 2 times the ULN value was considered as hepatotoxicity. |
| **Brito 2014** | Criteria for the diagnosis of anti-TB drug-induced hepatitis was an elevation in liver function tests, AST and/or ALT of more than 3-fold the ULN (reference values: 40 and 65 U/L, respectively) and/or total bilirubin up to >2.0 mg/dL in the presence of gastrointestinal symptoms such as anorexia, nausea, vomiting and/or jaundice, with serum ALT level normalisation after anti-TB drug discontinuation. |
| **Chamorro 2013** | Hepatotoxicity was defined as when serum transaminase concentrations were at least 3 times the ULN (normal values: AST between 0–32 IU/L and ALT between 0–31 IU/L) with report of jaundice (bilirubin normal values: 0–1 mg/dL) and/or hepatitis symptoms (nausea, vomiting, abdominal pain), or more than 5 times the ULN with or without symptoms. |
| **Cho 2007, Lee 2010** | ATDH was designated as an increase in serum ALT level more than 2 times the ULN value after anti-TB treatment, according to the criteria of drug-induced liver injuries developed by the international consensus meeting [1]. |
| **Feng 2014, Teixeira 2011,** | Anti-TB drug-induced hepatitis (Teixeira 2011)/Anti-TB drug-induced hepatic injury (Feng 2014): An increase in serum transaminase values to higher than 3 times the ULN values (40 IU/L ALT in Feng) and symptoms compatible with hepatitis. |
| **Fernandes 2015, Santos 2013 (both GI: SANTOS)** | Hepatotoxicity was defined as an increase in serum ALT level more than 3 times the ULN after treatment. |
| **Fredj 2016** | The causality of drug-induced hepatotoxicity was determined according to the report of an international consensus meeting [1]. These criteria include: (i) an increase of liver transaminases levels of more than 2 times above the normal value (<40 UI per litre) for AST and ALT; (ii) an improvement of this pattern after the drug withdrawal; and (iii) the absence of alternative causes of this disorder. |
| **Gogtay 2016** | Those patients with symptoms/signs such as anorexia, nausea, vomiting, malaise, icterus, and raised serum aminotransferase levels more than 2 times the ULN value or more than 5 times the ULN without clinical symptoms were considered to have hepatotoxicity. |
| **Gupta 2013**  **(GI: GUPTA)** | Increase in ALT over 2 times of ULN or a combined increase in AST and bilirubin levels, provided one of them is above 2 times of ULN, was defined as ATDH according to the international consensus meeting [1]. |
| **He 2015** | ATLI was defined according to the Danan criteria promulgated in 1990 [1,2]. No further information was provided. |
| **Huang 2003** | Anti-TB drug-induced hepatitis was diagnosed as: (i) an increase in serum ALT level greater than twice the ULN during treatment, according to the criteria established by the international consensus meeting [1]; (ii) negative serum hepatitis B virus surface antigen, IgM antibody to hepatitis A virus, and antibody to hepatitis C virus when ALT or AST is elevated; (iii) without any other major hepatic or systemic diseases that may induce elevation of liver biochemical tests, such as alcoholic liver disease, autoimmune hepatitis, congestive heart failure, hypoxia, and bacteraemia; and (iv) a causality assessment score greater than 5 (when classified as “probable” or “highly probable” drug-induced hepatitis), as derived from the international consensus meeting [1]. |
| **Kim 2009 (GI: KIM)** | Anti-TB drug-induced hepatitis was defined as an elevation in the serum levels of ALT above 2-times the ULN range (≤40 U/mL) during treatment and normalisation of these values after cessation of medication according to the criteria from the international consensus meeting [1]. |
| **Rana 2014** | ATDH was defined according to international consensus criteria [1]. Patients with a rise in serum AST or ALT levels more than or equal to 5 times of ULN, irrespective of symptoms and serum bilirubin levels, or patients with rise in serum AST or ALT levels more than or equal to 2 times of ULN with hyperbilirubinaemia and an absence of serological evidence of infection with hepatitis viruses (A, B, C and E) were considered as having ATDH. |
| **Roy 2006** | Definition was not reported. |
| **Sharma 2014** | ATDH was diagnosed if any one of criteria (i), (ii) or (iii) were present along with criteria (iv) and (v). The criteria were: (i) an increase of 5 times the ULN (50 international units [IU]/l) of serum AST and/or ALT levels on one occasion or more than 3 times (>150 IU/l) on 3 consecutive occasions; (ii) serum total bilirubin level >1.5 mg/dl; (iii) any increase in serum AST and/or ALT above pre-treatment values, together with anorexia, nausea, vomiting and jaundice; (iv) absence of serological evidence of infection with hepatitis viruses A, B, C or E; and (v) improvement in liver function (serum bilirubin <1 mg/dl, AST and ALT <100 IU/l) after the withdrawal of anti-TB drugs. |
| **Singla 2014** | International consensus criteria [1] define ATDH as development of more than 2 times the ULN value of ALT and AST. The ULN values used in this study were 35 U/L ALT and 40 U/L AST. |
| **Sotsuka 2011** | The severity of hepatotoxicity (hepatotoxicity A-D) was judged by the increase in either AST or ALT levels from the ULN range (AST, 33 U/L; ALT, 42 U/L): hepatotoxicity A, above the upper limit and less than 2-fold increase; hepatotoxicity B, 2- to 3-fold increase; hepatotoxicity C, 3- to 4-fold increase; hepatotoxicity D, greater than 4-fold increase. Results for grades B-D of hepatotoxicity were used in this review as clinical opinion was that the hepatotoxicity A patients would not have met the criteria for hepatotoxicity in many of the other studies included in this review. |
| **Tang 2012, Tang 2013a, Tang 2013b (all GI: ADACS)** | ATDH was defined as: (i) an increase to over 2 times the ULN in ALT or a combined increase in AST and total bilirubin, provided one of them was more than 2 times above the ULN; (ii) causality was assessed as certain, probable or possible based on the WHO Uppsala Monitoring Center criteria [3]. |
| **Wang 2010** | The selection criteria for ATDH were as follows: (i) ALT ≥2 times the ULN; (ii) increased AST/ALT/serum proteins (i.e. liver damage based on an increase in ALT or bilirubin ≥2 times the ULN, or an increase in AST, alkaline phosphatase and total bilirubin with at least one of these being ≥2 times the ULN); (iii) negative for hepatitis A antibody, hepatitis B surface antigen and hepatitis C marker; (iv) no other factors influencing the levels of AST/ALT/serum proteins, such as alcohol-induced liver disease, hypoxia, auto-immune disease, congestive heart failure and bacteraemia; and (v) causality assessment score >5. |
| **Wang 2011** | Among patients with normal baseline liver function (including AST, ALT, and total bilirubin), HATT was defined as increased serum AST and/or ALT >3 times the ULN in symptomatic, or >5 times the ULN in asymptomatic patients. Among those with increased baseline AST and/or ALT, HATT was defined as increased serum AST and/or ALT >1.5 times the baseline level. |
| **Xiang 2014** | ATLI was defined as an ALT, AST or bilirubin value more than 2 times the ULN value. The ULN used in the study was 40 U/L for ALT, 40 U/L for AST, and 19 mmol/L for total bilirubin. |
| **Yamada 2009** | ATDH was defined as an increase in serum AST level more than 2 times the ULN during 9 months of treatment with INH according to the criteria of the international consensus meeting in Paris [1]; normalisation of serum AST level after discontinuation of isoniazid; and a causality assessment score [4] of greater than 8, corresponding to the category of highly probable hepatotoxicity. |
| **Zaverucha-do-Valle 2014** | Hepatotoxicity was defined as a 2-fold increase in the normal upper limit (ALT 42 IU/L) or at least a 2-fold increase in ALT initial levels for those patients with a baseline ALT of >84 IU/L, during the treatment period. |

ALT: alanine aminotransferase; AST: aspartate aminotransferase; ATDH: anti-tuberculosis drug-induced hepatotoxicity; ATLI: anti-tuberculosis drug-induced liver injury; DILI: drug-induced liver injury; GI: group identifier; HATT: hepatitis during anti-tuberculosis treatment; INH: isoniazid; TB: tuberculosis; ULN: upper limit of normal

1. Benichou C. Criteria of drug-induced liver disorders. Report of an international consensus meeting. J Hepatol. 1990;11(2):272-6.

2. Dufour DR, Lott JA, Nolte FS, Gretch DR, Koff RS, Seeff LB. Diagnosis and monitoring of hepatic injury. I. Performance characteristics of laboratory tests. Clin Chem. 2000;46(12):2027-49.

3. World Health Organization. The use of the WHO-UMC system for standardized case causality assessment. Uppsala: The Uppsala Monitoring Centre. 2005:2-7.

4. Danan G, Benichou C. Causality assessment of adverse reactions to drugs—I. A novel method based on the conclusions of international consensus meetings: application to drug-induced liver injuries. J Clin Epidemiol. 1993;46(11):1323-30.
