# Supplementary material for: CYP genetic variants and toxicity related to anti-tubercular agents: a systematic review and meta-analysis
Source: Syst Rev. 2018 Nov 20;7:204. doi: 10.1186/s13643-018-0861-z (PMC6247669; doi:10.1186/s13643-018-0861-z)
Supplement: Supplementary file 5 — Table S3. Definitions of other toxicity outcomes. (DOCX 15 kb) [file 13643_2018_861_MOESM5_ESM.docx]

| **Outcome** | **Study** | **Outcome definition** |
| --- | --- | --- |
| **Adverse DIH outcome** | Bose (2011) | “16 [patients] showed an adverse outcome of ATT hepatotoxicity with icterus, severe nausea, and vomiting”. No further details reported. |
| **ADR** | Costa (2012) | The presence of at least one of the following symptoms during the follow-up period: gastric, joint, neuromuscular, or skin reactions; and hepatotoxicity (in accordance with the criteria of drug-induced liver injuries developed by the international consensus meeting)[1]. |
| **ATD-induced MPE** | Kim (2011)  (GI: KIM) | The development of MPE after receiving first-line ATD and the disappearance of MPE after discontinuing ATD because of MPE. |

**Additional file 5: Table S3. Definitions of other toxicity outcomes.**

ADR: adverse drug reaction; ATD: anti-tuberculosis drug; ATT: anti-tuberculosis treatment; DIH: drug-induced hepatotoxicity; GI: group identifier; MPE: macropapular eruption.

1. Saukkonen JJ, Cohn DL, Jasmer RM, Schenker S, Jereb JA, Nolan CM, et al. An official ATS statement: hepatotoxicity of antituberculosis therapy. Am J Respir Crit Care Med. 2006;174(8):935-52.
