# Supplementary material for: CYP genetic variants and toxicity related to anti-tubercular agents: a systematic review and meta-analysis
Source: Syst Rev. 2018 Nov 20;7:204. doi: 10.1186/s13643-018-0861-z (PMC6247669; doi:10.1186/s13643-018-0861-z)
Supplement: Supplementary file 6 — Results of the sensitivity analyses. (DOCX 62 kb) [file 13643_2018_861_MOESM6_ESM.docx]

**Additional file 6: Results of the sensitivity analyses.**

**Sensitivity analysis 1: Pairwise comparisons for the *CYP2E1* *Rsa*I polymorphism.**

*Heterozygous genotype (CT)* versus *homozygous wild-type (CC).*

**Fig S1. *CYP2E1* *Rsa*I polymorphism and anti-tuberculosis drug-induced hepatotoxicity: heterozygous genotype (CT) versus homozygous wild-type (CC).**

CI: confidence interval; GI: group identifier; OR: odds ratio; WT: wild-type.

*Homozygous mutant-type (TT)* versus *homozygous wild-type (CC).*

**

**Fig S2. CYP2E1 RsaI polymorphism and anti-tuberculosis drug-induced hepatotoxicity: homozygous mutant-type (TT) versus homozygous wild-type (CC).**

CI: confidence interval; GI: group identifier; MT: mutant-type; OR: odds ratio; WT: wild-type.

**Sensitivity analysis 2: Pairwise comparisons for the *CYP2E1 Dra*I polymorphism.**

*Heterozygous genotype (AT) versus homozygous wild-type (TT).*

**

**Fig S3. CYP2E1 DraI polymorphism and anti-tuberculosis drug-induced hepatotoxicity: heterozygous genotype (AT) versus homozygous wild-type (TT).**

CI: confidence interval; GI: group identifier; OR: odds ratio; WT: wild-type.

*Homozygous mutant-type (AA)* versus *homozygous wild-type (TT).*

**Fig S4. CYP2E1 DraI polymorphism and anti-tuberculosis drug-induced hepatotoxicity: homozygous mutant-type (AA) versus homozygous wild-type (TT).**

CI: confidence interval; GI: group identifier; MT: mutant-type; OR: odds ratio; WT: wild-type.

**Sensitivity analysis 3: Pairwise comparisons for the *CYP2E1 Pst*I polymorphism.**

*Heterozygous genotype (CG) versus homozygous wild-type (GG).*

**

**Fig S5. CYP2E1 PstI polymorphism and anti-tuberculosis drug-induced hepatotoxicity: heterozygous genotype (CG) versus homozygous wild-type (GG).**

CI: confidence interval; GI: group identifier; OR: odds ratio; WT: wild-type.

*Homozygous mutant-type (CC)* versus *homozygous wild-type (GG).*

**

**Fig S6. CYP2E1 PstI polymorphism and anti-tuberculosis drug-induced hepatotoxicity: homozygous mutant-type (CC) versus homozygous wild-type (GG).**

CI: confidence interval; GI: group identifier; MT: mutant-type; OR: odds ratio; WT: wild-type.
