# Supplementary material for: CYP genetic variants and toxicity related to anti-tubercular agents: a systematic review and meta-analysis
Source: Syst Rev. 2018 Nov 20;7:204. doi: 10.1186/s13643-018-0861-z (PMC6247669; doi:10.1186/s13643-018-0861-z)
Supplement: Supplementary file 7 — Funnel plots for the primary analyses. (DOCX 20 kb) [file 13643_2018_861_MOESM7_ESM.docx]

**Additional file 7: Funnel plots for the primary analyses.**

CYP2E1 Rsa*I polymorphism*.

**Fig S1. Funnel plot for the analysis of CYP2E1 RsaI polymorphism and anti-tuberculosis drug-induced hepatotoxicity.**

CYP2E1 Dra*I polymorphism.*

**Fig S2. Funnel plot for the analysis of CYP2E1 DraI polymorphism and anti-tuberculosis drug-induced hepatotoxicity.**

CYP2E1 Pst*I polymorphism.*

**

**Fig S3. Funnel plot for the analysis of CYP2E1 PstI polymorphism and anti-tuberculosis drug-induced hepatotoxicity.**
