# Supplementary material for: CYP genetic variants and toxicity related to anti-tubercular agents: a systematic review and meta-analysis
Source: Syst Rev. 2018 Nov 20;7:204. doi: 10.1186/s13643-018-0861-z (PMC6247669; doi:10.1186/s13643-018-0861-z)
Supplement: Supplementary file 8 — CYP genetic variants and hepatotoxicity meta-analyses. (DOCX 38 kb) [file 13643_2018_861_MOESM8_ESM.docx]

**Additional file 8: *CYP* genetic variants and hepatotoxicity meta-analyses.**

**Pairwise comparisons for *CYP2E1* 96-bp (deletion-insertion SNP).**

*Heterozygous genotype (DI) versus homozygous wild-type (DD).*

**Fig S1. *CYP2E1* 96-bp SNP and hepatotoxicity: heterozygous genotype (DI) versus homozygous wild-type (DD).**

CI: confidence interval; GI: group identifier; OR: odds ratio; WT: wild-type.

*Homozygous mutant-type (II) versus homozygous wild-type (DD).*

**Fig S2. *CYP2E1* 96-bp SNP and hepatotoxicity: homozygous mutant-type (II) versus homozygous wild-type (DD).**

CI: confidence interval; GI: group identifier; MT: mutant-type; OR: odds ratio; WT: wild-type.

**Pairwise comparisons for *CYP2C9* rs4918758.**

*Heterozygous genotype (TC) versus homozygous wild-type (TT).*

**Fig S3. *CYP2C9* rs4918758 and hepatotoxicity: heterozygous genotype (TC) versus homozygous wild-type (TT).**

One of the studies (Kim 2009 [GI: KIM]) reports WT to be C and MT to be T, but the other study (Tang 2013b [GI: ADACS]), and the data, suggest that WT is T and MT is C.

CI: confidence interval; GI: group identifier; OR: odds ratio; WT: wild-type.

*Homozygous mutant-type (CC) versus homozygous wild-type (TT).*

**Fig S4. *CYP2C9* rs4918758 and hepatotoxicity: homozygous mutant-type (CC) versus homozygous wild-type (TT).**

One of the studies (Kim 2009 [GI: KIM]) reports WT to be C and MT to be T, but the other study (Tang 2013b [GI: ADACS]), and the data, suggest that WT is T and MT is C

CI: confidence interval; GI: group identifier; MT: mutant-type; OR: odds ratio; WT: wild-type.

**Pairwise comparisons for *CYP2B6* rs3745274.**

*Heterozygous genotype (GT) versus homozygous wild-type (GG).*

**Fig S5. *CYP2B6* rs3745274 and hepatotoxicity: heterozygous genotype (GT) versus homozygous wild-type (GG).**

CI: confidence interval; GI: group identifier; OR: odds ratio; WT: wild-type.

*Homozygous mutant-type (TT) versus homozygous wild-type (GG).*

**Fig S6. *CYP2B6* rs3745274 and hepatotoxicity*:* homozygous mutant-type (TT) versus homozygous wild-type (GG).**

CI: confidence interval; GI: group identifier; MT: mutant-type; OR: odds ratio; WT: wild-type.
