# Supplementary material for: CYP genetic variants and toxicity related to anti-tubercular agents: a systematic review and meta-analysis
Source: Syst Rev. 2018 Nov 20;7:204. doi: 10.1186/s13643-018-0861-z (PMC6247669; doi:10.1186/s13643-018-0861-z)
Supplement: Supplementary file 9 — Table S4. Summary of results for other toxicity outcomes. (DOCX 16 kb) [file 13643_2018_861_MOESM9_ESM.docx]

**Additional file 9: Table S4. Summary of results for other toxicity outcomes.**

| **Outcome** | **Gene** | **Variant** | **Study** | **Country** | **Ethnicity** | **Comparison** | **OR (95% CI)** | **# cases** | **# controls** |
| --- | --- | --- | --- | --- | --- | --- | --- | --- | --- |
| **Adverse DIH outcome** | *CYP2E1* | 7632 T-A/*Dra*I polymorphism (rs6413432) | Bose (2011) | India | NR | Hom MT (AA) or het (TA) vs hom WT (TT) | 0.40 (0.13, 1.23) | 16 | 202 |
| **ADR** | *CYP2E1* | 7632 T-A/*Dra*I polymorphism (rs6413432) | Costa (2012) | Brazil | 84% Black/mixed race, 16% other | Het (TA) vs hom WT (TT) | Data excluded^a^ | | |
|  |  |  |  |  |  | Hom MT (AA) vs hom WT (TT) | Data excluded^a^ | | |
|  |  | 1293 G-C/*Pst*I polymorphism (rs3813867) | Costa (2012) | Brazil | 84% Black/mixed race, 16% other | Het (GC) vs hom WT (GG) | Data excluded^a^ | | |
|  |  |  |  |  |  | Hom MT (CC) vs hom WT (GG) | Data excluded^a^ | | |
| **ATD-induced MPE** | *CYP2E1* | 1053 C-T/*Rsa*I polymorphism (rs2031920)^b^ | Kim (2011)  (GI: KIM) | South Korea | NR | Hom MT (TT) or het (CT) vs hom WT (CC) | 0.87 (0.46, 1.63) | 60 | 153 |
|  |  | -352 A-G (rs2070672) | Kim (2011)  (GI: KIM) | South Korea | NR | Hom MT (GG) or het (AG) vs hom WT (AA) | 1.02 (0.53, 1.96) | 61 | 155 |
|  |  | -333 A-T (rs2070673) | Kim (2011)  (GI: KIM) | South Korea | NR | Hom MT (AA) or het (TA) vs hom WT (TT) | 1.12 (0.61, 2.03) | 61 | 154 |
|  | *CYP2C9* | -1188 C-T (rs4918758) | Kim (2011)  (GI: KIM) | South Korea | NR | Hom MT (CC) or het (TC) vs hom WT (TT) | 0.95 (0.50, 1.81) | 60 | 156 |
|  |  | -1565 C-T  (rs9332096) | Kim (2011)  (GI: KIM) | South Korea | NR | Hom MT (TT) or het (CT) vs hom WT (CC) | 0.23 (0.07, 0.78) | 61 | 157 |
|  |  | I359L  (rs1057910) | Kim (2011)  (GI: KIM) | South Korea | NR | Hom MT (CC) or het (AC) vs hom WT (AA) | 1.31 (0.47, 3.68) | 60 | 154 |
|  | *CYP2C19* | W212X (rs4986893) | Kim (2011)  (GI: KIM) | South Korea | NR | Hom MT (AA) or het (GA) vs hom WT (GG) | 0.30 (0.10, 0.88) | 61 | 157 |
|  |  | -1418 C-T | Kim (2011)  (GI: KIM) | South Korea | NR | Hom MT (TT) or het (CT) vs hom WT (CC) | 0.57 (0.27, 1.23) | 59 | 156 |

ATD: anti-tuberculosis drug; ADR: adverse drug reaction; CI: confidence interval; DIH: drug-induced hepatotoxicity; GI: group identifier; Het: heterozygous genotype; Hom MT: homozygous mutant-type; Hom WT: homozygous wild-type; MPE: maculopapular eruption; NR: not reported; OR: odds ratio.

^a^ Data excluded due to zero counts.

^b^ The study (Kim 2011 [GI: KIM]) refers to this SNP as -1055C>T.
